# Piperlongumine overcomes osimertinib resistance via governing ubiquitination-modulated Sp1 turnover

**DOI:** 10.1172/jci.insight.186165

**Published:** 2025-03-24

**Authors:** Ruirui Wang, Qiang Wang, Jinzhuang Liao, Xinfang Yu, Wei Li

**Affiliations:** 1 Department of Radiology and; 2 NHC Key Laboratory of Translational Research on Transplantation Medicine, Department of Transplant Surgery, The Third Xiangya Hospital of Central South University, Changsha, China.; 3 Key Laboratory of Carcinogenesis and Cancer Invasion of Chinese Ministry of Education, Xiangya Hospital, Central South University, Changsha, China.; 4 Key Laboratory of Carcinogenesis of National Health Commission, Cancer Research Institute and School of Basic Medical Science, Xiangya School of Medicine, Central South University, Changsha, China.

**Keywords:** Cell biology, Oncology, Lung cancer

## Abstract

Non–small cell lung cancer (NSCLC) is a common cause of cancer-related deaths worldwide, and its incidence has been increasing in recent years. While targeted therapies like osimertinib, an epidermal growth factor receptor tyrosine kinase inhibitor, have brought about notable improvements in patient outcomes for advanced NSCLC, the challenge of acquired drug resistance persists. Here, we found that cellular mesenchymal-epithelial transition factor (c-Met) was highly expressed in osimertinib-resistant cells, and depletion of c-Met markedly inhibited the growth of osimertinib-resistant cells ex vivo and in vivo, suggesting that c-Met is a potential target to address osimertinib resistance. Through a screening process using a natural product compound library, we identified piperlongumine as a potent inhibitor to overcome osimertinib resistance. Furthermore, the combined treatment of piperlongumine and osimertinib exhibited robust antitumor effects in resistant cells, partially restoring their sensitivity to osimertinib. Additionally, we discovered that piperlongumine could enhance the interaction between E3 ligase RNF4 and Sp1, inhibit the phosphorylation of Sp1 at Thr739, facilitate the ubiquitination and degradation of Sp1, lead to c-Met destabilization, and trigger intrinsic apoptosis in resistant cells. In summary, our study sheds light on the potential of piperlongumine in overcoming osimertinib resistance, offering new strategies and perspectives for the clinical management of drug-resistant NSCLC.

## Abstract 2

Piperlongumine overcomes osimertinib resistance via governing ubiquitination-modulated Sp1 turnover in non-small cell lung cancer

## Introduction

Lung cancer remains the leading cause of cancer deaths worldwide, with non–small cell lung cancer (NSCLC) representing the most common histopathologic type. The standard treatments for NSCLC include surgery, radiotherapy, biologically targeted therapy, and immunotherapy (1, 2). With the development of precision medicine and biotechnology, molecularly targeted therapies have greatly improved the outcome of patients with NSCLC (3, 4). One such target is the epidermal growth factor receptor (EGFR), a transmembrane protein highly expressed in NSCLC, making it a crucial therapeutic target for the disease (5, 6). Research indicates that tyrosine kinase inhibitors (TKIs) represent a promising therapeutic approach for patients with advanced NSCLC harboring EGFR mutations (5). EGFR TKIs can reversibly and selectively inhibit the activity and phosphorylation of EGFR-related tyrosine kinases by competitively binding to ATP sites on the structural domains of the intracellular tyrosine kinases, thus inhibiting EGFR downstream signaling, accelerating apoptosis, antagonizing angiogenesis, inhibiting tumor metastasis, and blocking tumor growth (7, 8). Osimertinib, an irreversible third-generation EGFR TKI, has become the first-line standard-of-care strategy for patients with advanced NSCLC harboring EGFR-activating mutations (9, 10). However, like other EGFR TKIs, osimertinib inevitably generates acquired resistance (11, 12). Studies have shown that the resistance mechanisms of osimertinib are mainly classified into 3 categories: EGFR-dependent resistance mechanisms, including EGFR mutations (e.g., C797X) and EGFR copy number amplification; activation of the bypass alternative pathway, including mesenchymal-epithelial transition factor (MET) amplification, human epidermal growth factor receptor 2 (HER2) amplification, PI3K catalytic subunit alpha (PIK3CA) mutation, phosphatase and tensin homolog deletion, B-Raf proto-oncogene (BRAF) mutation, and KRAS mutation; and phenotypic transformation, including epithelial-mesenchymal transition (EMT), pathological type transformation, tumor microenvironment remodeling, and metabolic reprogramming. The diversity of these resistance mechanisms makes single-targeted therapy challenging and highlights the impact of tumor heterogeneity on treatment outcomes. Consequently, an in-depth investigation of the intrinsic mechanisms of osimertinib resistance is essential for developing new targeted agents to overcome resistance and improve the prognosis of patients with NSCLC carrying EGFR-activating mutations.

The receptor tyrosine kinase cellular Met (c-Met) exerts multiple regulatory functions integral to various cellular processes, including the modulation of cell survival, proliferation, and EMT during migration. These functions are pivotal in mammalian development, tissue maintenance, and repair (13–16). In addition, extensive research has established a correlation between dysregulated c-Met and the progression of various cancers, such as colorectal, lung, cervical, hepatocellular, and head and neck cancers (17–22). Furthermore, reports indicate that the c-Met signaling pathway is vital in EGFR TKI-acquired resistance (23, 24). Therefore, c-Met could be a promising therapeutic target for patients with osimertinib-resistant NSCLC, providing a broad research prospect for the clinical treatment of NSCLC.

Specificity protein 1 (Sp1), as a transcription factor, plays a pivotal role in tumorigenesis by regulating the tumor cell cycle, apoptosis, DNA damage response, angiogenesis, and tumor migration and invasion (25). Studies have confirmed that Sp1 regulates the expression of c-Met by enhancing its transcription, thereby promoting tumor cell proliferation, migration, and drug resistance, substantially influencing tumorigenesis and progression (26–28). For example, in hepatocellular carcinoma (HCC), the deubiquitinase USP33 stabilizes Sp1, leading to increased c-Met expression and enhanced HCC metastasis (29). Basha et al. demonstrated that tolfenamic acid inhibits ovarian cancer (OC) cell growth in vitro and in vivo by disrupting Sp1-mediated regulation of c-Met, and they suggested that targeting c-Met by Sp1 protein degradation may be a promising strategy for OC therapeutic applications (30). In addition, Fang et al. showed that in NSCLC, the combination treatment of the herbal soup FZKA Tang and erlotinib inhibited and reciprocally regulated DNA methyltransferase and Sp1 to reduce the expression of MET genes, which resulted in the inhibition of NSCLC cell growth (31). These studies revealed the crucial role of Sp1 and c-Met in tumor development and their potential to overcome resistance to EGFR TKIs. Therefore, the interaction of Sp1 and c-Met may play a substantial role in osimertinib-resistant NSCLC. This study will delve into their mechanisms of action and provide molecular targets for treating NSCLC.

Piperlongumine, an alkaloid derived from the pepper plant species, exhibits a diverse range of biological activities, including the regulation of lipid metabolism and antiplatelet agglutination, and exhibits antiinflammatory, antimicrobial, analgesic, antidiabetic, and antitumor properties (32). Its antitumor effects encompass inhibiting tumor cell cycle progression, attenuating angiogenesis and invasive metastasis, and regulating tumor energy metabolism (33, 34). Recent studies have highlighted its potent antitumor activity against various cancers, including lung cancer (35), breast cancer (36), colorectal cancer (37), and HCC (38). Notably, piperlongumine can inhibit NSCLC cell growth, induce NSCLC cell pyroptosis, and effectively reverse NSCLC resistance to cisplatin through multiple signaling pathways (39–43). However, the mechanism of its effect on osimertinib-resistant NSCLC cells and the potential molecular targets remains unclear, necessitating further in-depth studies.

In this study, we explored the mechanism of piperlongumine’s effect in osimertinib-resistant NSCLC cells, and our report indicated that the Sp1/c-Met axis plays a critical role in promoting osimertinib resistance in NSCLC cells. This finding offers the possibility of a new therapeutic strategy for NSCLC.

## Results

### c-Met plays a crucial role in tumorigenicity of osimertinib-resistant NSCLC cells.

**Figure 1:**
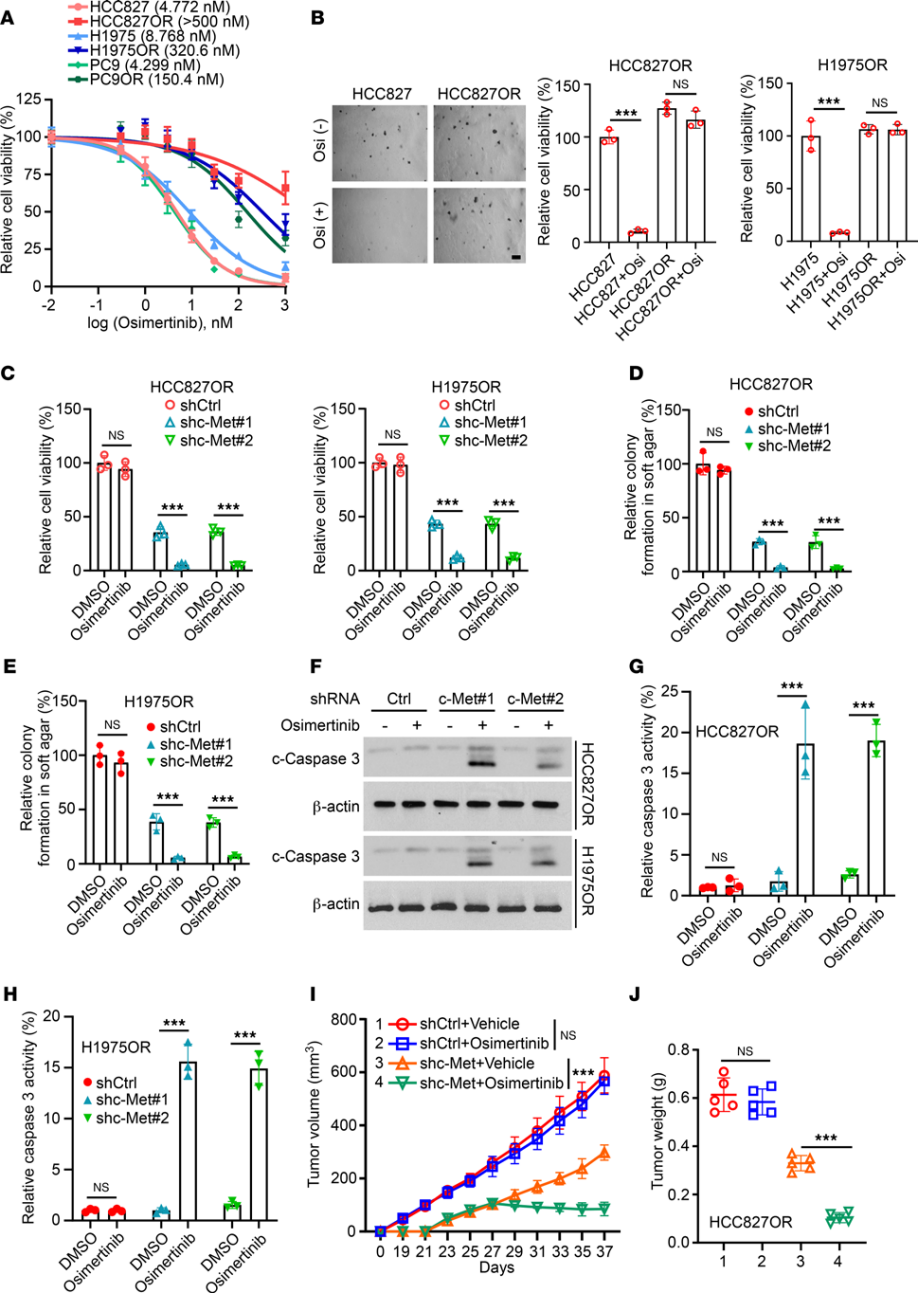
Silencing of c-Met inhibits the malignant phenotype of osimertinib-resistant cells. (**A**) MTS assay was performed after treating HCC827/HCC827OR, H1975/H1975OR, and PC9/PC9OR cells with osimertinib for 24 hours. (**B**) Colony-forming ability of HCC827/HCC827OR and H1975/H1975OR cells was detected using soft agar assay after treatment with osimertinib. Scale bar, 200 μm. ***P < 0.001. (**C**–**H**) Stable cell lines with c-Met gene silencing were constructed with HCC827OR and H1975OR cells and given osimertinib or DMSO treatment. Cell viability was analyzed by MTS assay (**C**, *n* = 3. ***P < 0.001), colony formation ability was determined by soft agar assay (**D** and **E**, *n* = 3, ***P < 0.001), protein expression level of c-caspase-3 was analyzed by immunoblotting assay (**F**), and activity level of caspase-3 was detected by caspase-3 activity assay kit (**G** and **H**, *n* = 3, ***P < 0.001). (**I** and **J**) shc-Met HCC827OR cells were utilized to construct a xenograft transplantation tumor model and analyze the effects of c-Met deletion and administration of osimertinib treatment on tumor volume (**I**, ***P < 0.001) and tumor weight (**J**, *n* = 5, ***P < 0.001). Comparisons were performed by using 1-way ANOVA test (**B**–**E** and **G**–**J**). Data are presented as the mean ± SD (**B**–**E**, **G**, and **H**).

Previous reports have highlighted a strong correlation between the c-Met signaling pathway and NSCLC resistance to osimertinib (23, 24). To gain insight into the specific mechanism of c-Met in NSCLC osimertinib resistance, we established osimertinib-resistant cell lines using H1975 and PC9 as parental cell lines. Initially, we generated 7 clones of H1975 and PC9 cells in constructing osimertinib resistance cell lines and analyzed them with the lysate of their parental cells by Western blotting (WB). The results showed that the expression of c-Met in clone 7 was upregulated most obviously (Supplemental Figure 1, A and B; supplemental material available online with this article; https://doi.org/10.1172/jci.insight.186165DS1). Therefore, we selected the No. 7 resistant clone cell line for the follow-up experiment. To verify the resistance of these cells, we used HCC827OR, H1975OR, and PC9OR to assess cell viability and colony formation ability following osimertinib treatment. The results showed that the IC_50_ values of osimertinib-resistant cells were substantially higher than the parental osimertinib-sensitive cells after osimertinib treatment (Figure 1A) and that there was no marked difference in the ability of drug-resistant cells to form cell colonies after osimertinib treatment (Figure 1B and Supplemental Figure 1C). Furthermore, immunofluorescence (IF) results showed that there was also no marked difference in the expression of phospho–histone H3 Ser10 and proliferating cell nuclear antigen (PCNA), a marker of cell proliferation, after osimertinib treatment in resistant cells (Supplemental Figure 1, D and E). Subsequently, we established stable cell lines with c-Met gene silencing in osimertinib-resistant HCC827OR and H1975OR cells (Supplemental Figure 1F). Our results showed that silencing of c-Met substantially inhibited the cell viability and colony formation of HCC827OR and H1975OR cells (Supplemental Figure 1, G and H), and this effect was further enhanced after cotreatment with osimertinib (Figure 1, C–E, and Supplemental Figure 2, A and B). In addition, our results revealed that silencing of c-Met substantially increased the protein expression of cleaved caspase-3 (c-caspase-3) and the activity of caspase-3 in osimertinib-resistant NSCLC cells (Figure 1, F–H). These observations suggest that c-Met affects the sensitivity of NSCLC to osimertinib. Next, we established a xenograft tumor model using short hairpin RNA control (shCtrl) and short hairpin RNA c-Met (shc-Met) HCC827OR cells. The results illustrated that c-Met deletion decreased tumor volume (Supplemental Figure 1I), tumor size (Supplemental Figure 1J), and tumor weight (Supplemental Figure 1K), and these effects were further enhanced after treatment with osimertinib (Figure 1, I and J, and Supplemental Figure 2C). Moreover, the survival time of tumor-bearing mice was prolonged (Supplemental Figure 1L). These data indicated that c-Met is essential for maintaining the tumorigenicity of osimertinib-resistant NSCLC cells.

### Piperlongumine inhibits osimertinib-resistant NSCLC cells.

**Figure 2:**
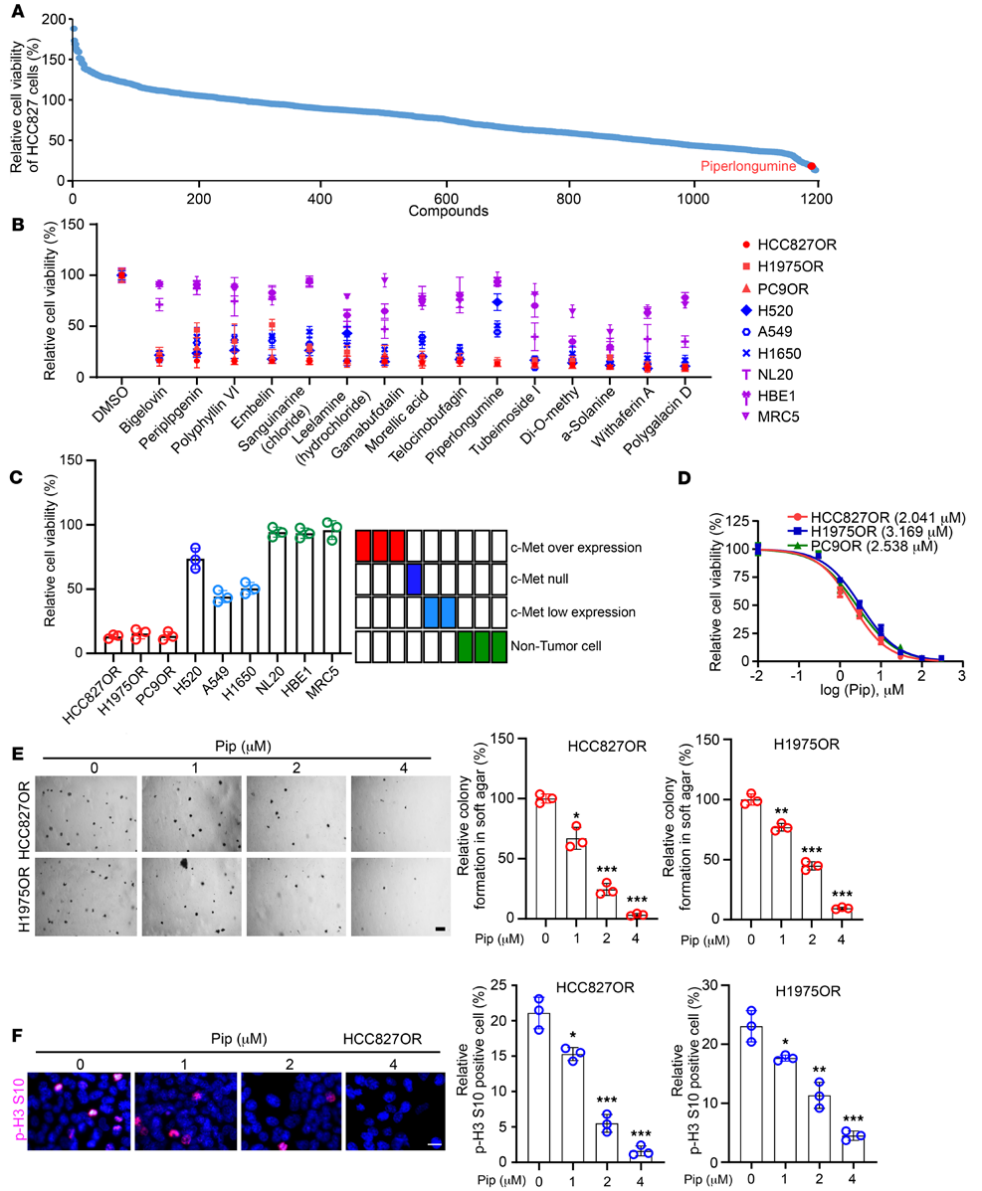
Piperlongumine inhibits osimertinib-resistant NSCLC cell lines. (**A**) Cell viability was measured by MTS to screen for natural compounds that effectively inhibited HCC827OR cells. The red dot labeling represents piperlongumine. (**B**) MTS assay determined the effect of different compounds, including piperlongumine, on the cell viability of HCC827OR, H1975OR, PC9OR, H520, A549, H1650, NL20, HBE1, and MRC5 cells. (**C**) MTS assay analysis of the effect of piperlongumine on the viability of HCC827OR, H1975OR, PC9OR, H520, A549, H1650, NL20, HBE1, and MRC5 cells. (**D**) HCC827OR, H1975OR, and PC9OR cells were treated with different concentrations of piperlongumine; MTS assay was then performed to detect the cell viability. (**E** and **F**) HCC827OR and H1975OR cells were treated with different concentrations of piperlongumine. The colony-forming ability of piperlongumine was detected by soft agar assay after piperlongumine treatment for 24 hours (**E**, scale bar, 200 μm. **P < 0.01. ***P < 0.001), and the phosphorylation level of histone H3 Ser10 in resistant cells was analyzed by immunofluorescence (IF) after piperlongumine treatment for 24 hours (**F**, scale bar, 10 μm. *P < 0.05. **P < 0.01. ***P < 0.001). Comparisons were performed using 1-way ANOVA test (**E** and **F**, *n* = 3). Data are presented as the mean ± SD (**E** and **F**).

We then screened a library of natural products (1,197 compounds, Supplemental Table 1) to discover small molecule compounds that could overcome osimertinib resistance. The top 15 compounds (including bigelovin, periplogenin, polyphyllin VI, embelin, sanguinarine, leelamine, gamabufotalin, morellic acid, telocinobufagin, tubeimoside I, Di-O-methyldemethoxycurcumin, α-solanine, withaferin A, and polygalacin D) with the greatest inhibitory effects against HCC827OR were identified (Figure 2A). Next, we tested the inhibitory effects of these 15 natural products on a panel of immortalized nontumor cells (NL20, MRC5, and HBE1) and lung cancer cells with high protein level of c-Met (HCC827OR, H1975OR, and PC9OR), medium level of c-Met (H1650 and A549), or null c-Met (H520). Cell viability results indicate that only piperlongumine substantially inhibited the viability of HCC827OR, H1975OR, and PC9OR cells (c-Met overexpression) while having no marked impact on NL20, HBE1, and MRC5 cells. It also exhibits impaired inhibitory effects on the viability of A549 and H1650 cells (c-Met low expression) and is further compromised in H520 cells (Figure 2, B and C). These results indicated that piperlongumine substantially inhibited the growth of osimertinib-resistant NSCLC cells, which was associated with c-Met expression. Subsequently, we treated HCC827OR, H1975OR, and PC9OR cells with different concentrations of piperlongumine, resulting in IC_50_ values of 2.041 μM, 3.169 μM, and 2.538 μM, respectively (Figure 2D). Based on these results, we selected treatment concentrations of piperlongumine as 0 μM, 1 μM, 2 μM, and 4 μM for subsequent experiments. Our findings demonstrated dose-dependent inhibition of colony formation and proliferation in resistant cells by piperlongumine (Figure 2, E and F, and Supplemental Figure 3A). Overall, the above data suggest that piperlongumine has the potential to inhibit osimertinib-resistant NSCLC cells.

### Piperlongumine promotes intrinsic apoptosis in osimertinib-resistant NSCLC cells.

**Figure 3:**
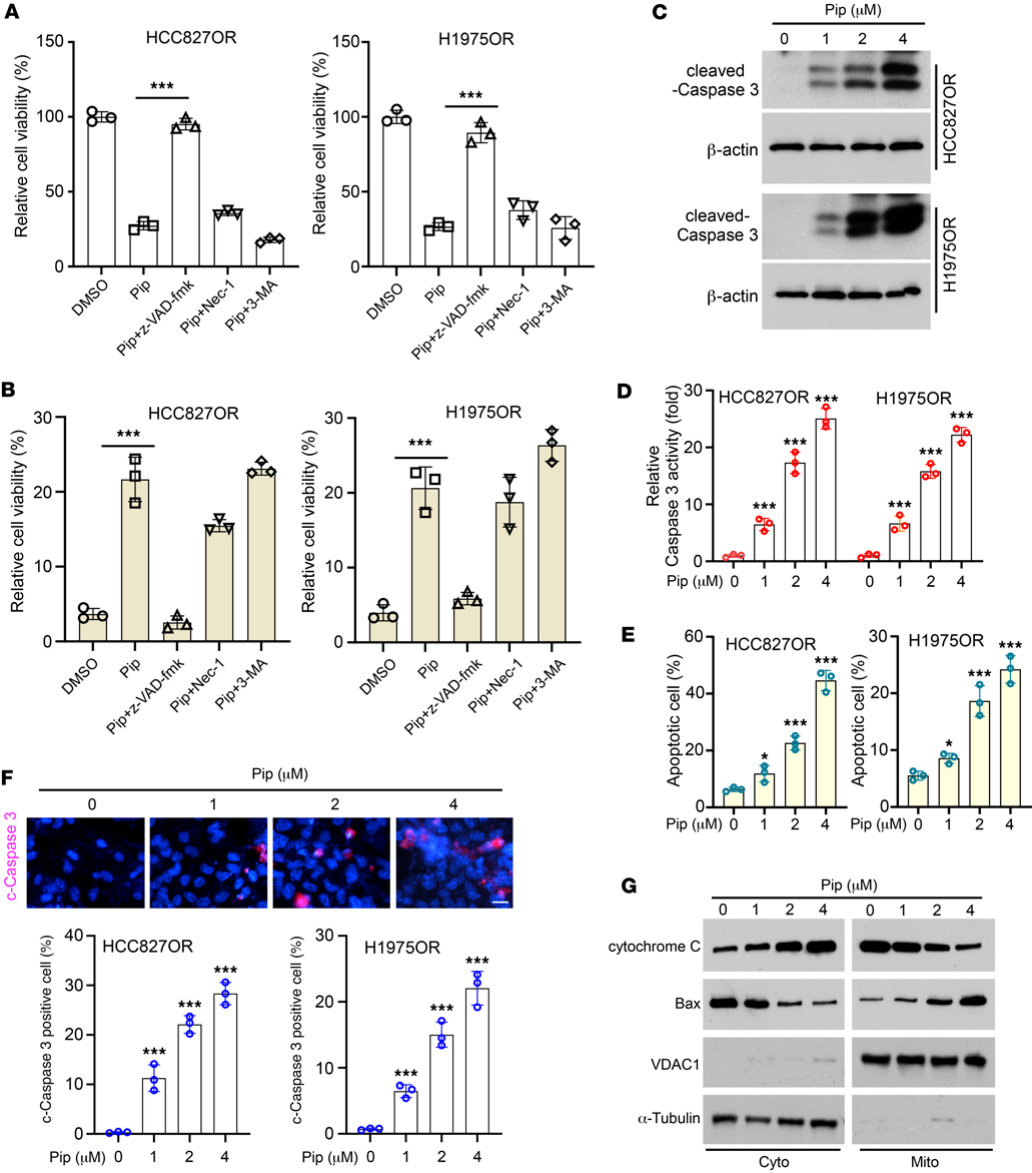
Piperlongumine promotes intrinsic apoptosis in osimertinib-resistant NSCLC cells. (**A** and **B**) HCC827OR and H1975OR cells were pretreated with Z-VAD-FMK, necrostatin-1, or 3-MA for 4 hours, followed by piperlongumine treatment for 24 hours. Cell viability was analyzed by the MTS assay (**A**), and the number of dead cells was counted by trypan blue staining and statistical analysis (**B**). ***P < 0.001. (**C**–**F**) After treating HCC827OR and H1975OR cells with different concentrations of piperlongumine for 24 hours, the protein expression level of c-caspase-3 was detected by immunoblotting (IB) (**C**), the activity level of caspase-3 was detected by caspase-3 activity assay kit (**D**, ***P < 0.001), and the level of apoptosis was detected by flow cytometry (**E**, *P < 0.05. ***P < 0.001). The expression level of c-caspase-3 was detected by IF (**F**, scale bar, 10 mm. ***P < 0.001). (**G**) After treatment of HCC827OR cells with different concentrations of piperlongumine for 24 hours, subcellular fractions were isolated for IB analysis. Comparisons were performed by using 1-way ANOVA test (**A**, **B**, and **D**–**F**, *n* = 3). Data are presented as the mean ± SD (**A**, **B**, and **D**–**F**).

To investigate how piperlongumine inhibits osimertinib-resistant NSCLC cells, we examined whether piperlongumine induced cell death. We pretreated HCC827OR and H1975OR cells with inhibitors Z-VAD-FMK (apoptosis), necrostatin-1 (necroptosis), and 3-MA (autophagy). We observed a substantial restoration of cell viability (Figure 3A) and reduced dead cells (Figure 3B) only when piperlongumine was coadministered with the apoptosis inhibitor Z-VAD-FMK. Subsequently, we pretreated HCC827OR and H1975OR cells with piperlongumine and detected the protein expression levels of c-caspase-3 (Figure 3C), the activity of caspase-3 (Figure 3D), the apoptosis level of cells (Figure 3E), and the number of c-caspase-3–positive cells (Figure 3F). All of these results indicated that piperlongumine treatment increased the apoptosis of osimertinib-resistant cells. In addition, we further evaluated the subcellular localization of apoptotic molecules, revealing that piperlongumine promoted the release of cytochrome *c* from the mitochondria to the cytoplasm while facilitating the translocation of Bcl-2-associated X protein (Bax) to the mitochondria (Figure 3G). These results demonstrated that piperlongumine induces endogenous apoptosis in osimertinib-resistant NSCLC cells.

### Piperlongumine exerts antitumor effects in osimertinib-resistant NSCLC cells through the Sp1/c-Met axis.

**Figure 4:**
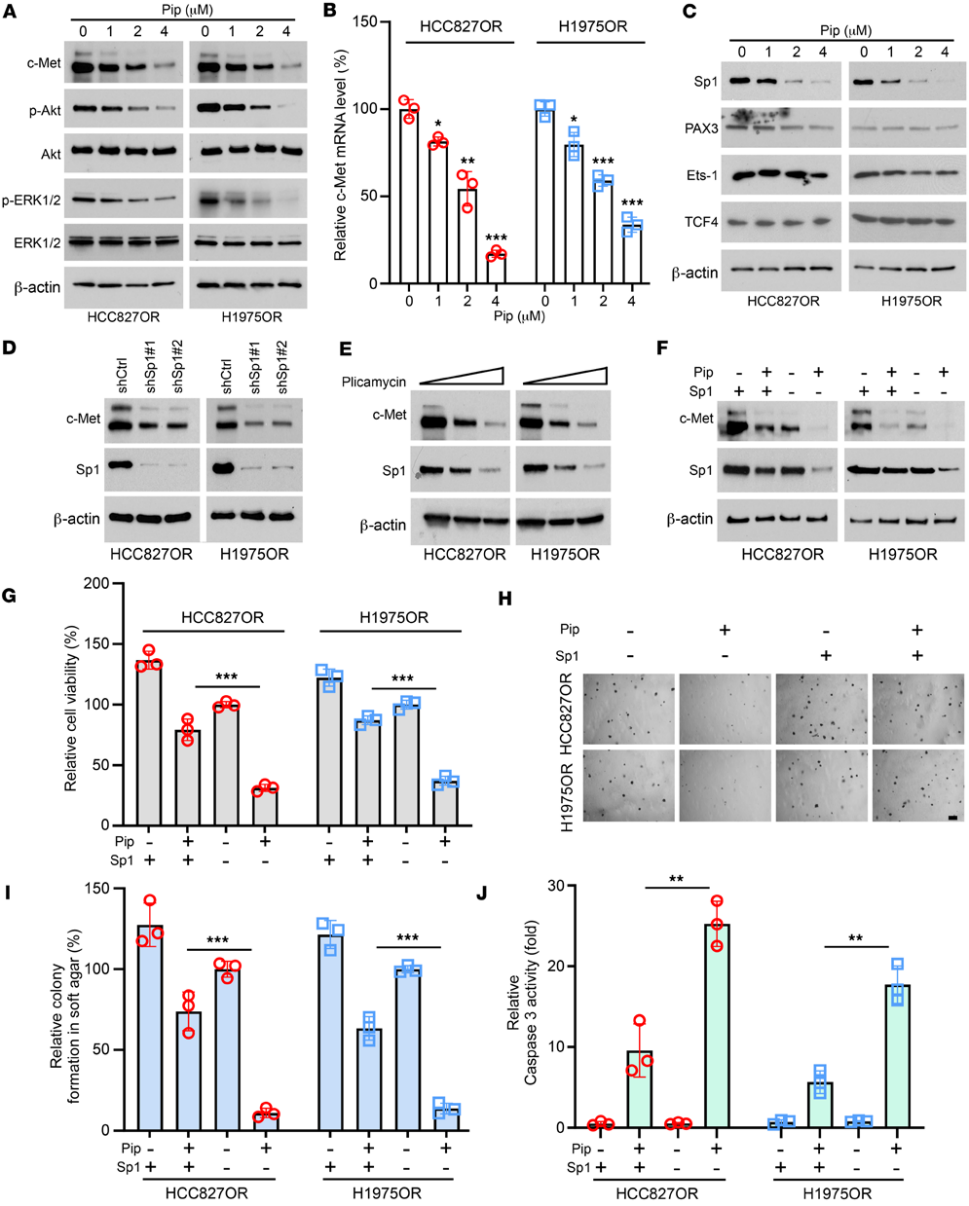
Piperlongumine inhibits osimertinib-resistant cells by inhibiting the Sp1/c-Met axis. (**A**–**C**) After treating HCC827OR and H1975OR cells with different concentrations of piperlongumine for 24 hours, cells were collected and subjected to IB (**A** and **C**) and quantitative PCR analysis (**B**, *P < 0.05. **P < 0.01. ***P < 0.001). (**D**) Sp1 gene-silenced stable cell lines were constructed using HCC827OR and H1975OR cells, and protein expression levels of c-Met and Sp1 were analyzed by IB. (**E**) HCC827OR and H1975OR cells were treated with different doses of plicamycin, and whole-cell lysates (WCE) were collected and subjected to IB analysis. (**F**–**J**) Sp1 was overexpressed in HCC827OR and H1975OR cells and treated with piperlongumine. c-Met and Sp1 protein expression levels were analyzed by IB (**F**), cell viability was determined by MTS assay (**G**, ***P < 0.001), colony-forming ability was determined by soft agar assay (**H** and **I**, scale bar, 200 mm. ***P < 0.001), and caspase-3 activity assay kit detected caspase-3 activity (**J**, **P < 0.01). Comparisons were performed using 1-way ANOVA (**B**, **G**, **I**,and **J**, *n* = 3). Data are presented as the mean ± SD (**B**, **G**, **I**, and **J**).

We further investigated the impact of piperlongumine on the c-Met signaling pathway by treating HCC827OR and H1975OR cells with different concentrations of piperlongumine. Immunoblotting revealed a dose-dependent decrease in the protein levels of c-Met, phosphorylated ERK1/2 (p-ERK1/2), and p-Akt. In contrast, the total protein levels of Akt and ERK1/2 remained unchanged (Figure 4A). Furthermore, the quantitative reverse transcription PCR (qRT-PCR) data showed that the mRNA expression levels of c-Met in HCC827OR and H1975OR cells were negatively correlated with the piperlongumine concentration (Figure 4B), suggesting that piperlongumine influences protein expression by regulating c-Met transcription. Therefore, we examined the impact of piperlongumine treatment on the c-Met transcription–related transcription factors E26 transformation-specific sequence-1 (Ets-1), paired box gene 3 (PAX3), Sp1, and T cell factor 4 (TCF4). Our data demonstrated that piperlongumine downregulated the protein expression levels of Sp1. At the same time, no marked effect was observed on the protein expression levels of Ets-1, PAX3, and TCF4 (Figure 4C). Moreover, our experimental results showed that prolonged piperlongumine treatment substantially inhibited Sp1 and c-Met over extended periods (Supplemental Figure 4A), suggesting its potential for long-term therapeutic effects. To further elucidate the role of Sp1 in c-Met expression in osimertinib-resistant cells, stable cell lines with Sp1 knockdown were established. Immunoblotting results showed that the protein expression level of c-Met was subsequently reduced after Sp1 knockdown (Figure 4D). Similarly, the expression of c-Met also decreased in a dose-dependent manner after treatment with the Sp1 inhibitor plicamycin (Figure 4E), suggesting that Sp1 regulates c-Met transcription in osimertinib-resistant cells. Furthermore, ectopic overexpression of Sp1 restored the protein expression level of c-Met, cell viability, and colony formation ability in piperlongumine-treated, drug-resistant cells (Figure 4, F–I). In addition, Sp1 overexpression decreased caspase-3 activity in piperlongumine-treated HCC827OR and H1975OR cells (Figure 4J). These results indicate that piperlongumine inhibits c-Met expression by repressing the transcription factor Sp1.

### Piperlongumine enhances RNF4-mediated Sp1 ubiquitination and degradation.

**Figure 5:**
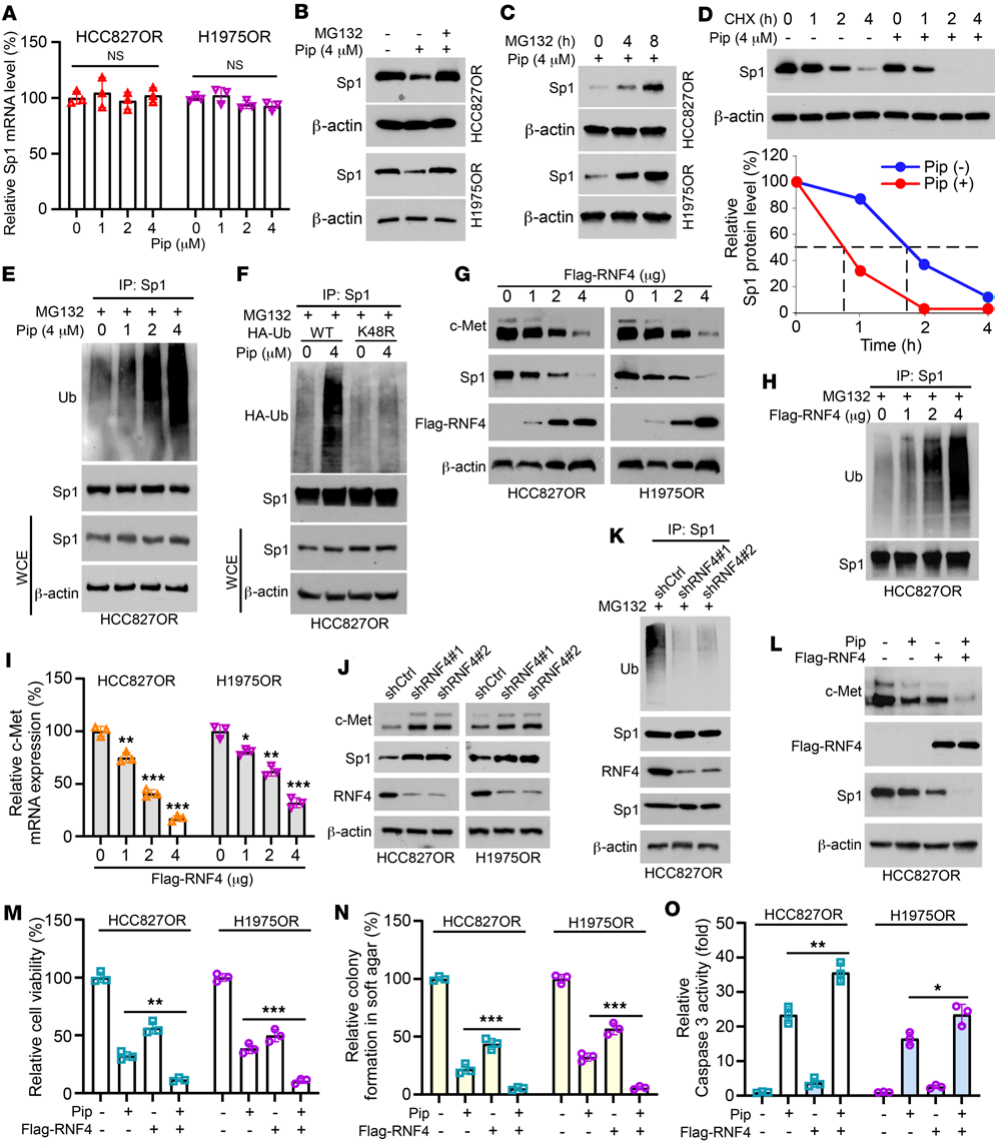
Piperlongumine promotes ubiquitination and degradation of Sp1. (**A**) HCC827OR and H1975OR cells were treated with different concentrations of piperlongumine for 24 hours, and Sp1 mRNA levels were analyzed by qRT-PCR. (**B**) HCC827OR and H1975OR cells were treated with 4 μM piperlongumine for 24 hours, followed by 6 hours of MG132 (20 μM), and WCE was analyzed by IB. (**C**) HCC827OR and H1975OR cells were treated with 4 μM piperlongumine for 24 hours, followed by MG132 treatment for different times (0, 4, 8 hours), and WCE was analyzed by IB. (**D**) HCC827OR cells were treated with or without 4 μM piperlongumine for 24 hours, followed by cycloheximide (CHX) (20 μg/mL) treatment, and WCE was analyzed by IB. (**E**) HCC827OR cells were treated with different concentrations of piperlongumine for 24 hours, followed by MG132 (20 μM) for 8 hours, and ubiquitylation was analyzed. (**F**) HA-Ub WT and K48R mutant plasmids were transfected into HCC827OR cells and treated with piperlongumine for 24 hours, and ubiquitylation was analyzed. (**G**) HCC827OR and H1975OR cells were transfected with Flag-RNF4 plasmids for 24 hours, and WCE was analyzed by IB. (**H**) Flag-RNF4–transfected cells were treated with MG132 for 8 hours and analyzed for ubiquitination. (**I**) The level of c-Met mRNA was analyzed by qRT-PCR after Flag-RNF4 transfection. *P < 0.05. **P < 0.01. ***P < 0.001. (**J** and **K**) RNF4 gene-silenced stable cell lines were established, and WCE was analyzed by IB. (**L**–**O**) Flag-RNF4–transfected cells were treated with piperlongumine, followed by WCE collection for IB analysis (**L**), and cell viability (**M**), colony formation (**N**), and caspase-3 activity (**O**) were analyzed. *P < 0.05. **P < 0.01. ***P < 0.001. Comparisons were performed by using 1-way ANOVA test (**A**, **I**, **M**–**O**, *n* = 3). Data are presented as the mean ± SD (**A**, **I**, **M**–**O**).

To clarify the specific mechanism of downregulation of Sp1 by compounds, we initially investigated the impact of piperlongumine on Sp1 mRNA levels in HCC827OR and H1975OR cells and found that piperlongumine did not substantially affect Sp1 mRNA expression (Figure 5A). However, we discovered that treatment with the proteasome inhibitor MG-132 led to a notable restoration of Sp1 protein expression, with a more pronounced effect observed with longer duration of MG-132 treatment (Figure 5, B and C). Furthermore, we observed a shortened half-life of Sp1 in drug-resistant cells after piperlongumine treatment (Figure 5D), indicating that piperlongumine might downregulate Sp1 protein level through the proteasomal degradation pathway. To investigate this hypothesis further, we examined the ubiquitination of Sp1 in osimertinib-resistant cells with piperlongumine treatment and found a dose-dependent increase in the ubiquitination of Sp1 in HCC827OR cells (Figure 5E). Subsequently, we transfected HCC827OR cells with the HA-Ub-WT/K48R (lysine mutated to arginine) mutant plasmid and found that HA-Ub-K48R mutant impaired Sp1 ubiquitination, indicating piperlongumine promotes Sp1 degradation (Figure 5F). Considering that RNF4 is a known ubiquitin E3 ligase for Sp1 (44), we transfected RNF4 into osimertinib-resistant cells and found a decrease in the protein levels of Sp1 and c-Met with increasing amounts of RNF4 transfection (Figure 5G). Moreover, RNF4 promotes Sp1 ubiquitination (Figure 5H) and decreases c-Met mRNA (Figure 5I). To further elucidate the mechanism of RNF4 action on Sp1 and c-Met, we established RNF4 gene-silenced stable cells using osimertinib-resistant HCC827OR and H1975OR cells. Our findings indicate that silencing the RNF4 gene led to an increase in the protein expression levels of Sp1 and c-Met, along with a subsequent decrease in the ubiquitination level of Sp1 (Figure 5, J and K). Additionally, RNF4 overexpression reduced the protein levels of c-Met and Sp1, cell viability, and colony formation ability in osimertinib-resistant cells, and these effects were further enhanced by piperlongumine treatment (Figure 5, L–N). Similarly, RNF4 overexpression enhanced the activity of caspase-3 (Figure 5O). These results suggest that piperlongumine promotes RNF4-mediated Sp1 ubiquitination and degradation.

### Piperlongumine reverses osimertinib resistance by destabilizing Sp1 via a phosphorylation-dependent mechanism.

**Figure 6:**
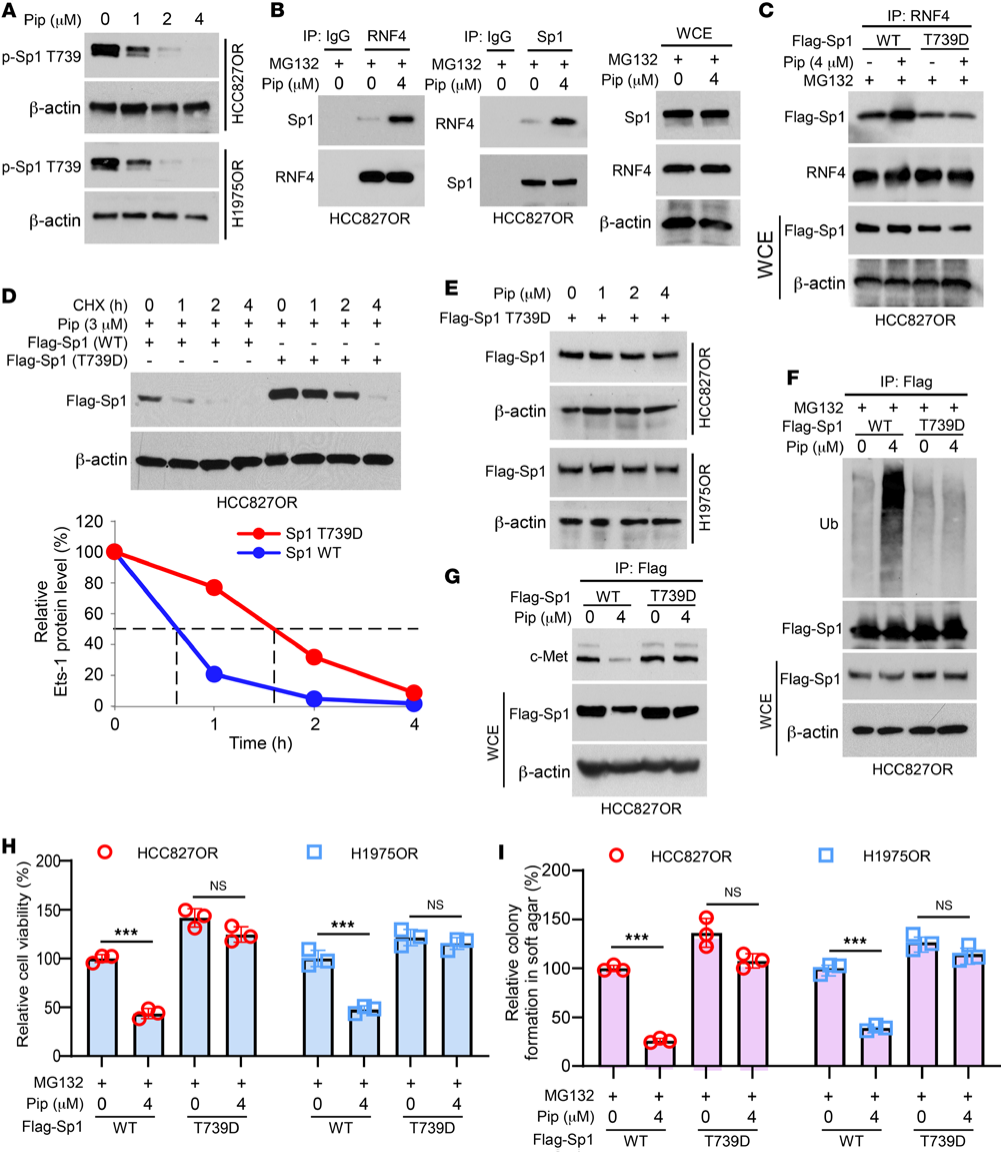
Piperlongumine destabilizes Sp1 in a Thr739 phosphorylation–dependent manner. (**A**) HCC827OR and H1975OR cells were treated with different concentrations of piperlongumine, and WCE was collected for IB analysis. (**B**) HCC827OR cells were treated with or without piperlongumine for 24 hours and after coincubation with MG-132 for 8 hours. Immunoprecipitation (IP) assay was performed to detect the interaction between RNF4 and Sp1. (**C**) Flag-Sp1-WT or -T739D was transfected into HCC827OR cells, followed by piperlongumine treatment for 24 hours. MG-132 was added to the medium and maintained for 8 hours. Cells were collected and IB analysis was performed. (**D**). The corresponding plasmids were transfected into HCC827OR cells. Piperlongumine treatment was given for 24 hours followed by treatment with CHX (20 μg/mL) for different time points. Cells were collected for IB analysis. (**E**) Flag-Sp1-T739D plasmid was transfected into HCC827OR and H1975OR cells overnight, followed by various doses of piperlongumine treatment for 24 hours. WCE was collected for IB analysis. (**F**) Flag-Sp1-WT, or -T739D, was transfected into osimertinib-resistant cells and treated with piperlongumine (4 μM) for 24 hours. MG-132 was added to the medium and maintained for 8 hours. Cells were collected for ubiquitination analysis. (**G**) Flag-Sp1-WT, or -T739D, was transfected into osimertinib-resistant cells and treated with piperlongumine (4 μM) for 24 hours. Cells were collected for IB analysis. (**H** and **I**) MTS (**H**) and the soft agar assay (**I**) determined cell viability and colony formation ability, respectively. ***P < 0.001. Comparisons were performed using 1-way ANOVA (**H** and **I**, *n* = 3). Data are presented as the mean ± SD (**H** and **I**).

The previous reports indicate that Sp1 phosphorylation can be regulated by a variety of kinases, including protein kinase C (45), ERK1/2 (46), cell cycle protein-dependent kinases (CDK1/2) (47), c-Jun NH_2_-terminal kinase (JNK) (48), casein kinase II (49), and AMP-activated protein kinase (50). It has been shown that the stability of Sp1 is primarily regulated by phosphorylation at 3 specific sites, Ser59, Thr453, and Thr739; that ERK1/2 is involved in the regulation of phosphorylation at these 3 sites; and that phosphorylation at the Thr739 site is also regulated by CDK1 (51, 52). Our data suggest that piperlongumine treatment promotes the ubiquitination and degradation of Sp1. We therefore examined the phosphorylation of ERK1/2 and CDK1 in osimertinib-resistant cells. We observed that the phosphorylation of ERK1/2 (p-ERK1/2 T202/Y204), CDK1 (T161), and Sp1 (T453 and T739) was upregulated in osimertinib-resistant cells (HCC827OR and H1975OR) (Supplemental Figure 5A). Notably, piperlongumine treatment inhibited the phosphorylation of ERK1/2, CDK1 (Supplemental Figure 5B), and Sp1 (T453 and T739) (Supplemental Figure 5C and Figure 6A). To further elucidate the role of ERK1/2 and CDK1 in the regulation of Sp1 in osimertinib-resistant cells, we treated the cells with specific inhibitors of ERK1/2 (PD98059), CDK1 (Ro-3306), or piperlongumine. The findings showed that inhibition of ERK1/2 and CDK1 substantially reduced the protein level of Sp1 and c-Met (Supplemental Figure 5D), suggesting that ERK1/2 and CDK1 are essential for regulating Sp1 expression in osimertinib-resistant cells. Moreover, phosphorylation of the Thr739 site plays a crucial role in regulating the protein stability and DNA binding activity of Sp1 (53–55). Previous reports have shown that phosphorylation of the Thr739 site prevents Sp1 from being degraded by RNF4-mediated ubiquitination, thereby maintaining its function in cells (44). Consistent with previous studies, our results showed that piperlongumine treatment enhanced the interaction between RNF4 and WT Sp1 (Figure 6B) but had no marked effect on Flag-Sp1-T739D (mimics phosphorylated sustained activation state) (Figure 6C). The results of cycloheximide (CHX) experiments showed that Flag-Sp1-T739D prolonged the half-life of Sp1 compared with Flag-Sp1-WT even in the presence of piperlongumine (Figure 6D). Similarly, Flag-Sp1-T739D restored the expression level of Sp1 in piperlongumine-treated osimertinib-resistant cells (Figure 6E). Moreover, piperlongumine failed to induce the ubiquitination of Flag-Sp1-T739D (Figure 6F) and restored the protein expression levels of Sp1 and c-Met (Figure 6G). In addition, cell viability assay and soft agar assay showed that Flag-Sp1-T739D substantially rescued cell viability and colony formation ability in piperlongumine-treated HCC827OR and H1975OR cells (Figure 6, H and I). These results indicate that ERK1/2 and CDK1 play a critical role in developing osimertinib resistance by regulating the phosphorylation of Sp1. Inhibiting these signaling pathways may provide a new therapeutic strategy for overcoming resistance. Furthermore, piperlongumine overcomes resistance by inhibiting Sp1 phosphorylation and promoting its degradation, further supporting its potential as a therapeutic strategy.

### Piperlongumine inhibits the in vivo tumorigenesis in osimertinib-resistant NSCLC cells.

**Figure 7:**
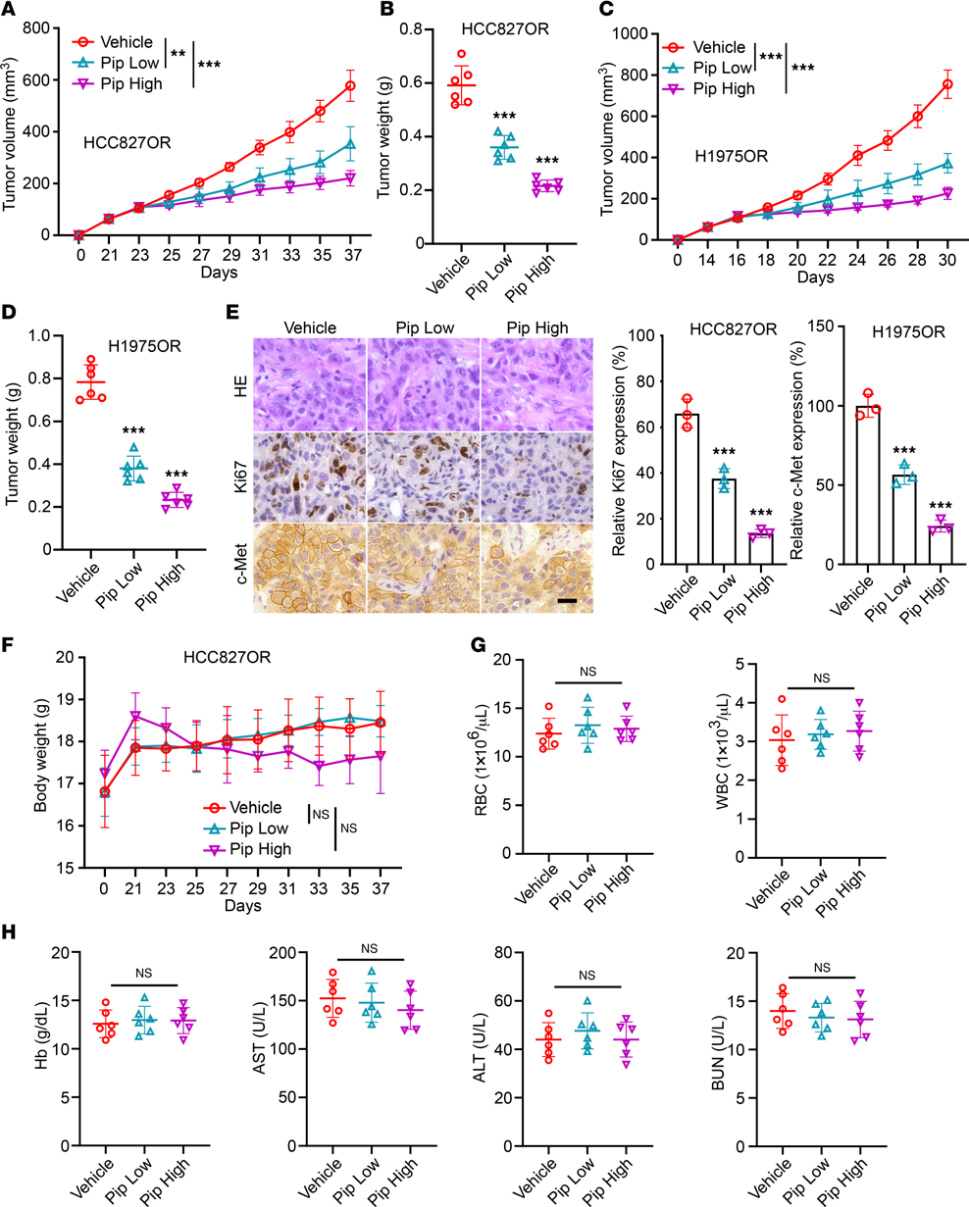
Piperlongumine inhibits the growth of osimertinib-resistant cells in vivo. (**A** and **B**) HCC827OR-derived xenograft tumors were subjected to different doses of piperlongumine, and the resulting tumor volume (**A**) and weight (**B**) were recorded. *n* = 6, **P < 0.01. ***P < 0.001. (**C** and **D**) Different concentrations of piperlongumine were administered to H1975OR xenograft tumors, and the subsequent measurements of tumor volume (**C**) and weight (**D**) were documented. ***P < 0.001. (**E**) HCC827OR-derived xenograft tumor tissues were subjected to IHC assay of Ki67 and c-Met. scale bar, 10 μm. ***P < 0.001. (**F**) The body weights of tumor-bearing mice after different doses of piperlongumine administration. (**G** and **H**) Mouse blood analysis following different doses of piperlongumine administration. Comparisons were performed by using 1-way ANOVA test (**A**–**H**, *n* = 6). Data are presented as the mean ± SD (**A**–**H**).

To assess the effect of piperlongumine treatment on NSCLC osimertinib-resistant cell lines in vivo, we constructed xenograft tumor models using HCC827OR and H1975OR cells. The data showed that tumor volume, size, and weight were more pronounced in the high-dose piperlongumine-treated group than in the low-dose group (Figure 7, A–D). Furthermore, immunohistochemical (IHC) results showed a more pronounced decrease in the expression levels of Ki67 and c-Met in the high-dose piperlongumine-treated group (Figure 7E). Moreover, we also observed no marked changes in the body weight of the tumor-bearing mice following piperlongumine treatment (Figure 7F). Similarly, analysis of blood samples showed that piperlongumine treatment had no substantial alterations on white blood cells (WBC), red blood cells (RBC), hemoglobin (Hb), aspartate aminotransferase (AST), alanine aminotransferase (ALT), and blood urea nitrogen (BUN) in mice (Figure 7, G and H). In addition, IHC results showed that piperlongumine treatment caused no obvious damage to the heart, liver, spleen, lungs, and kidneys (Supplemental Figure 6A). These data suggest that piperlongumine exhibits good tolerability and effectively inhibits tumor growth of osimertinib-resistant cells in vivo.

### Piperlongumine resensitizes osimertinib in vivo.

**Figure 8:**
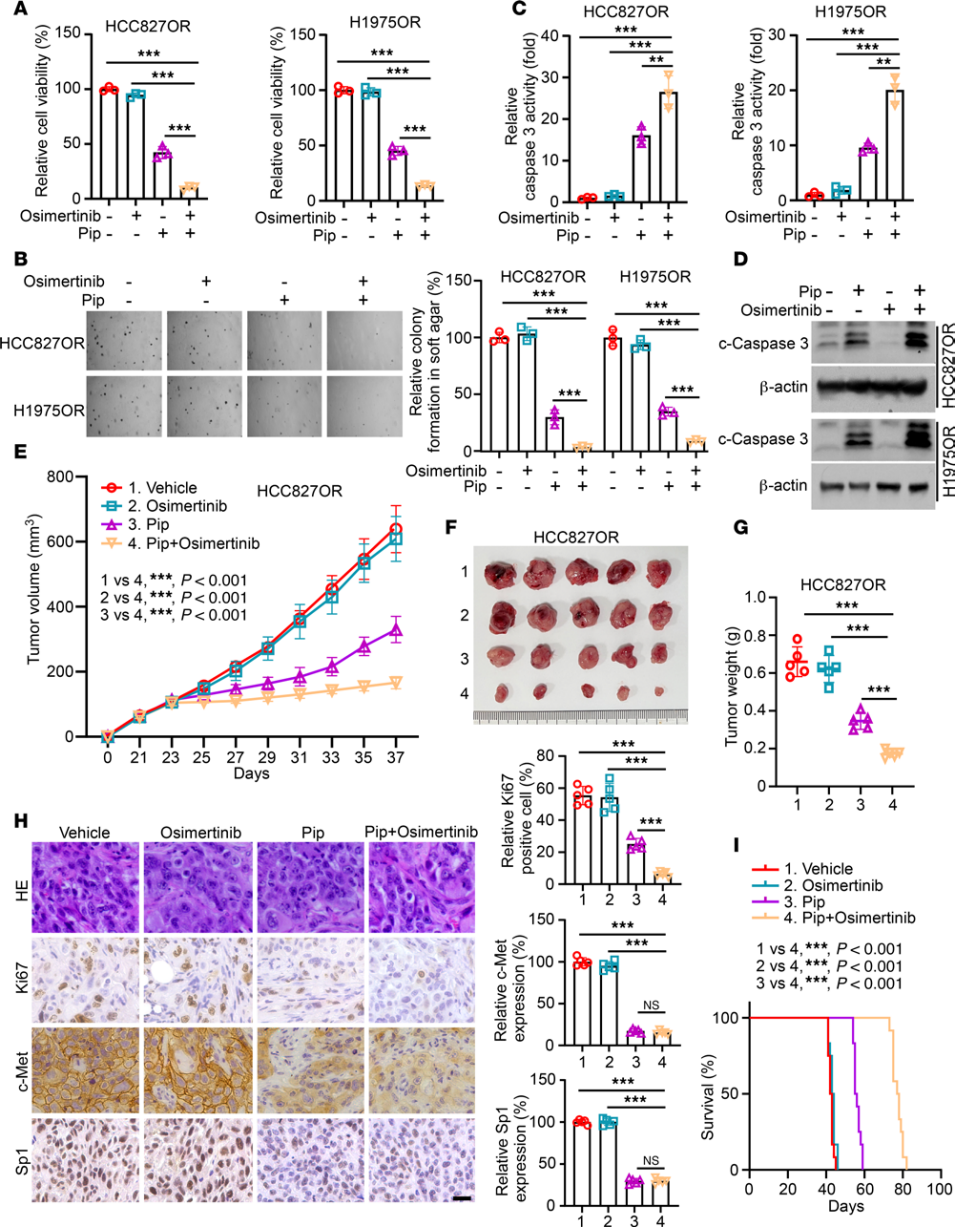
Piperlongumine restores the sensitivity of osimertinib-resistant NSCLC cells to osimertinib in vitro and in vivo. (**A**–**D**) HCC827OR and H1975OR cells were treated with piperlongumine, osimertinib, or a combination of both. Cell viability was analyzed by MTS assay (**A**, *n* = 3, ***P < 0.001), colony-forming ability was analyzed by soft agar assay (**B**, *n* = 3, scale bar, 200 μm. ***P < 0.001), caspase-3 activity was determined by caspase-3 activity assay kit (**C**, *n* = 3, **P < 0.01. ***P < 0.001), and the protein expression level of c-caspase-3 was detected by IB assay (**D**). (**E**–**I**) A xenograft tumor model was constructed using HCC827OR cells and treated with piperlongumine, osimertinib, or both in combination. Tumor volume (**E**), mass (**F**), and weight (**G**) were documented. *n* = 5, ***P < 0.001. IHC assay analysis of the expression levels of Ki67, c-Met, and Sp1 in the tumor tissues (**H**, scale bar, 10 μm. ***P < 0.001). The survival analysis of tumor-bearing mice with different treatments by Kaplan-Meier method (**I**). ***P < 0.001. Comparisons were performed by using 1-way ANOVA test (**A**–**C**, **E**, **G**, and **H**) and log-rank (Mantel-Cox) test (**I**). Data are presented as the mean ± SD (**A**–**C**, **E**, **G**, and **H**).

To investigate the potential of piperlongumine in resensitizing resistant NSCLC cells to osimertinib, we treated HCC827OR and H1975OR cells with piperlongumine. We found that piperlongumine decreased the cell viability (Figure 8A) and colony-forming ability (Figure 8B) of the resistant cells and increased the activity of caspase-3 and the c-caspase-3 protein expression levels (Figure 8, C and D). Moreover, the inhibitory efficacy was further enhanced when combined with osimertinib. Additionally, we observed that piperlongumine substantially suppressed tumor volume, size, and weight (Figure 8, E–G) and delayed in vivo tumorigenesis when cotreated with osimertinib. IHC results showed that the piperlongumine and osimertinib combination effectively downregulated the expression of Ki67, c-Met, and Sp1 in tumor tissues, leading to an extension in the survival time of mice (Figure 8, H and I). These results suggest that piperlongumine treatment can overcome the resistance of NSCLC cells to osimertinib in vivo.

## Discussion

Studies on the pathogenesis of NSCLC have delved deep into the realm of gene-molecule interactions, pointing toward a consensus that the development of NSCLC is a multifaceted process involving the orchestrated interplay of various genes and stages (56, 57). Notably, EGFR remains a focal point of investigation (58). The EGFR is localized on chromosome 7, contains 28 exons, and has tyrosine kinase activity. Previous studies have shown that TKIs are particularly effective in patients with activating mutations in the tyrosine kinase domain of the EGFR gene, underscoring TKIs as a promising therapeutic avenue for patients with advanced EGFR-mutated NSCLC (5). Despite the initial success of EGFR TKIs, reports indicate a common occurrence of resistance following prolonged treatment, posing a formidable challenge in patient management, especially with osimertinib, a potent, third-generation, irreversible EGFR TKI targeting EGFR mutations. The burgeoning research on osimertinib resistance in NSCLC has delineated 2 overarching categories of resistance mechanisms: EGFR-dependent (targeted) and EGFR-independent (off-target) mechanisms (12, 59–62). Among them, the incidence of osimertinib-targeted resistance is low, mainly including the C797X mutation interfering with drug-protein interactions (63, 64). The prevailing EGFR-independent resistance mechanisms encompass MET amplification, HER2 amplification, alterations in diverse tyrosine kinase receptors, signaling pathway modifications, cell cycle gene alterations, and histological transformation (62, 65–69). MET amplification is currently recognized as the most common EGFR-independent resistance mechanism, accounting for 5%–24% of osimertinib-resistant patients, with the MET oncogene encoding the c-Met receptor tyrosine kinase (62). Therefore, targeting MET/c-Met is of great importance for treating drug-resistant patients and devising effective therapeutic strategies for drug-resistant patients. Our study uncovered elevated c-Met expression in osimertinib-resistant cell lines, with c-Met knockdown substantially impeding the tumorigenicity of drug-resistant cells. Furthermore, xenograft tumor analysis revealed that c-Met deficiency extended the survival duration of mice, underscoring the potential prognostic value of c-Met in predicting osimertinib therapeutic sensitivity and highlighting it as a promising therapeutic target for combating drug resistance in NSCLC.

More importantly, c-Met overexpression was associated with genomic MET amplification and MET exon 14 mutations, in addition to transcription factors such as hypoxia-inducible factor 1α, FOXC2, PAX3, Sp1, TCF4, and Ets-1, which are involved in regulating c-Met expression and activation (29, 70–73). Although reports indicate many regulatory factors associated with c-Met overexpression, studies on transcription factors regulating c-Met expression levels in osimertinib-resistant cells have not been reported to our knowledge. Our findings indicate a positive correlation between the upregulation of c-Met expression and the transcription factor Sp1 in osimertinib-resistant cells, while no such association was observed with PAX3, TCF4, and Ets-1. Previous reports indicated that in hepatocellular carcinoma, esophageal carcinoma, lung cancer, and prostate cancer, the expression level of Sp1 was also correlated with the expression level of c-Met (29, 31, 74, 75). This suggests that targeting the Sp1/c-Met axis may provide a potential therapeutic strategy for osimertinib resistance. Furthermore, recent findings indicate that in colorectal cancer, c-Met in an acidic microenvironment promotes tumor metastasis and participates in immune escape from tumors by influencing the behavior and infiltration of immune cells, which may have implications for immunotherapy (76). A phase II study demonstrated that the combination of a MET inhibitor (capmatinib) with a programmed cell death 1 inhibitor (nivolumab) showed promising antitumor activity and a manageable safety profile in patients with advanced NSCLC with EGFR WT and unscreened programmed cell death ligand 1 (77). Notably, homoharringtonine (HHT) has been approved by the FDA for the treatment of chronic myeloid leukemia, with mechanistic studies suggesting that HHT may affect the DNA epitope by direct targeting of SP1 and by inhibiting the SP1-mediated transcriptional regulation of the *TET1* expression genome to exert antitumor effects (78). Additionally, Sp1 enhances immunosuppression in cholangiocarcinoma by regulating Snail expression and promoting EMT and immune escape (79). Therefore, targeting the Sp1/c-Met axis and acting synergistically with other therapeutic strategies is expected to greatly enhance the antitumor effect and open up new ideas for treating drug-resistant tumors. This integrated strategy not only overcomes the challenges posed by drug resistance but also provides new directions for future research, encouraging the exploration of more precisely targeted interventions in individualized therapies that may improve patient outcomes.

Sp1 protein turnover is regulated by ubiquitinases and deubiquitinases, such as USP39, USP33, and RNF4 (29, 44, 80). In the present study, we found an important interaction between the E3 ubiquitin ligase RNF4 and the transcription factor Sp1 and that RNF4 regulates the ubiquitination level of Sp1 in osimertinib-resistant cells. Specifically, gene silencing of RNF4 in osimertinib-resistant cells resulted in a marked increase in the expression of Sp1 and c-Met proteins and a decrease in the ubiquitination level of Sp1. This suggests that RNF4 regulates the protein levels of Sp1 by promoting its ubiquitinated degradation. We also examined the expression of ERK1/2 and CDK1 in osimertinib-resistant cells. The results showed that the phosphorylation of both ERK1/2 and CDK1 and the phosphorylation levels of Sp1 at Thr453 and Thr739 were substantially upregulated. Notably, treatment with piperlongumine substantially inhibited these phosphorylation changes. Further experiments demonstrated that inhibition of ERK1/2 and CDK1 led to a marked reduction in the protein levels of Sp1 and c-Met, suggesting that ERK1/2 and CDK1 play a crucial role in regulating Sp1 expression in osimertinib-resistant cells. Notably, Flag-Sp1-T739D reduced the ubiquitination level of Sp1 and markedly restored cell viability and clone formation ability in piperlongumine-treated resistant cells compared with Flag-Sp1-WT. This result suggests that Thr739 phosphorylation is involved in regulating Sp1 stability, but it also influences its sensitivity to ubiquitination degradation. Phosphorylation of Sp1 at the Thr739 site has been demonstrated to be essential for maintaining its protein stability and enhancing DNA binding activity (53–55), which further supports our findings. Additionally, other research has pointed out that RNF4 acts as a ubiquitin E3 ligase to promote Sp1 degradation. However, when Sp1 is phosphorylated by JNK at the Thr739 site, it prevents Sp1 from interacting with RNF4, thereby protecting Sp1 from degradation (44). Therefore, targeting Sp1 may be a potential strategy to overcome osimertinib resistance in NSCLC cells, and phosphorylation of Thr739 also plays a crucial role in this process.

Recently, there has been a growing focus on the antitumor properties of natural compounds derived from traditional Chinese medicine in tumor chemoprevention and treatment (81–83). Traditional Chinese medicines offer several advantages over conventional chemotherapeutic drugs, including low toxicity and cost-effectiveness (84–86). Recent studies have shown that the natural product piperlongumine exhibits potent antitumor effects by targeting multiple signaling pathways (39, 87, 88). For example, in esophageal squamous cell carcinoma, piperlongumine inhibited tumorigenesis by triggering NRF2/ROS/TXNIP/NLRP3-dependent pyroptosis (89). In addition, Sandeep et al. demonstrated that piperlongumine inhibits the proliferation of colon cancer cells by targeting Ras proteins and the PI3K/Akt signaling cascade, exhibiting marked anticancer properties (90). In leukemia studies, piperlongumine induced apoptosis and autophagy in leukemia cells by targeting ROS-p38/JNK signaling (91). Furthermore, piperlongumine plays a crucial role in overcoming chemotherapy resistance in tumors. For example, piperlongumine inhibited TRIM14 signaling by activating the p38/MAPK pathway, thereby enhancing the sensitivity of glioblastoma multiforme to the first-line chemotherapeutic agent temozolomide (92). Similarly, piperlongumine substantially enhanced the antitumor efficacy of oxaliplatin by inducing ROS in gastric cancer cells. These findings suggest the potential application of piperlongumine in tumor therapy (93). In the present study, we found that piperlongumine could target Sp1/c-Met and effectively inhibit the proliferation of osimertinib-resistant cells by enhancing the binding of RNF4 to Sp1. Importantly, our study demonstrated that piperlongumine did not exhibit substantial in vivo toxicity in a mouse xenograft model. Therefore, piperlongumine is expected to be a promising drug candidate to advance the research and application of cancer therapy.

Tumor cells often evade drug action through adaptive responses, mainly via feedback mechanisms, leading to the development of drug resistance (94–96). Wei et al. reported that the limited effect of AZD8055 as an ATP-competitive inhibitor of mTOR in pancreatic cancer cells was partly due to FOXO transcription factor–mediated upregulation of EGFR and its reactivation of Akt activity. Although AZD8055 was initially able to inhibit the activity of mTOR complexes 1/2 and the Akt signaling pathway, this inhibition was only transient; pancreatic cancer cells rapidly regained tolerance to the drug with the activation of EGFR and its downstream kinases (97). This finding suggests that tumor cells escape drug inhibition by crossactivating other signaling pathways, ultimately leading to treatment failure. Similar feedback mechanisms have been observed in other cancers and are closely linked to drug resistance. For example, in BRAF (V600E)–mutant thyroid cancer cells, treatment with the BRAF inhibitor PLX4032 was partially ineffective because of overexpression of c-Met, which in turn activated the PI3K/Akt and MAPK pathways (98). This is similar to the findings of Wei et al., suggesting that feedback mechanisms and cross-pathway activation play a critical role in tumor therapy. Combination therapy strategies are crucial to overcome this resistance. Wei et al. suggested that combining AZD8055 with erlotinib could substantially enhance the therapeutic effect by inhibiting the mTOR and EGFR pathways. This dual-targeted therapy effectively breaks the mechanism by which tumor cells escape drug inhibition through the EGFR/Akt pathway (97). Similarly, in BRAF inhibition therapy for thyroid cancer, the combination of a BRAF inhibitor (PLX4032) with a c-Met inhibitor (PHA665752) was also able to effectively inhibit the 2 major signaling pathways (MAPK and PI3K/AKT), thereby enhancing the therapeutic response and overcoming the resistance caused by a single inhibitor (98). These studies have shown that tumor cells can rapidly adapt to drug therapy and develop resistance through complex feedback regulation and crossactivation mechanisms. Therefore, combination therapy strategies can enhance the efficacy and effectively reduce the risk of drug resistance development. In our study, both in vivo and in vitro experiments demonstrated that piperlongumine substantially inhibited tumor growth and enhanced the therapeutic efficacy of osimertinib. However, considering the possible adaptive responses and drug resistance of tumor cells, future studies should focus on exploring and overcoming these feedback mechanisms to provide more personalized and durable treatment strategies for patients with different types of tumors.

In this study, piperlongumine, by targeting the Sp1/c-Met axis, is expected to overcome the resistance of NSCLC to osimertinib, and the combination of piperlongumine and osimertinib can enhance the sensitivity of NSCLC cells to osimertinib in vivo and vitro. Therefore, targeting the inactivation of the Sp-1/c-Met axis may be a promising strategy for the clinical management of NSCLC.

## Methods

### Sex as a biological variable.

In this study, sex was not considered as a biological variable in the animal experiments.

### Cell lines and cell culture.

NSCLC cell lines HCC827, H1975, PC9, H520, A549, and H1650 and immortalized nontumor cell lines NL20, HBE1, and MRC5 were purchased from the ATCC. These cells were cultured in a constant-temperature incubator (37°C and 5% CO_2_) with specific humidity under the guidance of ATCC protocols. The osimertinib-acquired resistance cell lines, H1975OR and PC9OR, were generated by exposing H1975 and PC9 cells to increasing concentrations of osimertinib for 6 months. HCC827OR cells with c-Met overexpression were a gift from Zigang Dong (The Hormel Institute, University of Minnesota, Austin, Minnesota, USA) (99).

### Reagents and antibodies.

Inhibitors involved in the study, including MG-132, CHX, necrostatin-1, Z-VAD-FMK, and 3-MA, were purchased from Selleck Chemicals. The natural compound library used in this study was procured from MedChemExpress. Antibodies against c-caspase-3 (REF.9664; IB: 1:1,000; IHC: 1:2,000), β-actin (REF.3700; IB: 1:1,000), c-Met (REF.8198; IB: 1:1,000; IHC: 1:200), cytochrome *c* (REF.11940; IB: 1:1,000), α-tubulin (REF.2125; IB: 1:5,000), Bax (REF.14796; IB: 1:1,000), VDAC1 (REF.4866; IB: 1:3,000), Akt (REF.4691; IB: 1:2,000), p-Akt (REF.4060; IB: 1:1,000), Ets-1 (REF.14069; IB: 2,000), p-ERK1/2 (REF.4370; IB: 1:1,000), ERK1/2 (REF.9102; IB: 1:2,000), PAX3 (REF.12412; IB: 1:1,000), TCF4 (REF.2569; IB: 1:1,000), Sp1 (REF.5931; IB:1:1,000), Ub (REF.3936; IB: 1:1,000), and Flag-tag (REF.8146; IB: 1:1,000) were obtained from Cell Signaling Technology, Inc. Lipofectamine 2000 transfection reagent (REF.11668019) and antibodies against RNF4 (REF.MA5-27423; IB: 1:1,000), p-Sp1 T739 (REF.PA5-104771; IB: 1:1,000), and p-Sp1 T453 (REF.PA5-104770; IB: 1:1,000) were purchased from Thermo Fisher Scientific. Antibodies against Ki67 (REF.ab15580; IHC: 1:2,000) and Sp1 (REF.ab124804; IHC: 1:2,000) were products of Abcam. Furthermore, shRNA plasmids used in this study, including c-Met shRNA (#1, TRCN0000040043; #2, TRCN0000000396), Sp1 shRNA (#1, TRCN0000020444; #2, TRCN0000020445), and RNF4 shRNA (#1, TRCN0000017053; #2, TRCN0000017054), were purchased from GE Horizon. cDNA plasmids, including Sp1 (SC101137) and RNF4 (RC207273), were obtained from Origene.

### MTS assay.

Cells were inoculated into 96-well plates at a density of 3 × 10^3^ and incubated at 37°C in a constant temperature incubator for 1 day, followed by treatment according to the experimental design. After treatment, MTS reagent (REF.G3581, Promega) was added to the 96-well plates and incubated for 1–2 hours at 37°C under light protection.

### Soft agar assay.

Cells were inoculated into 6-well plates at a density of 8 × 10^3^ cells/well. Subsequently, the cells were incubated in Eagle’s basal medium for 2 weeks (37°C) and colonies were observed. Finally, the number of colonies was counted by light microscopy.

### WB assay.

NSCLC cells were processed according to the experimental design, and the cell precipitate was collected by digestion and centrifugation at 500*g*, then added to a precooled RIPA cell lysis buffer (Thermo Fisher Scientific) to obtain WCE. The protein concentration was then determined using the BCA Protein Concentration Assay Kit (REF.22328) from Thermo Fisher Scientific. Protein samples of the same mass were separated on SDS-PAGE gels and transferred to PVDF membranes presoaked in methanol. Subsequently, the membrane was incubated in 5% skim milk for 1–2 hours. After sealing, precooled primary antibodies were added and incubated at 4°C overnight. The next day, the membrane was incubated with the corresponding secondary antibody for another 1 hour. Finally, exposure was performed using a chemiluminescence imager Amersham ImageQuant 800 (GE Healthcare).

### Caspase-3 activity assay.

NSCLC cells were collected and washed with PBS, followed by cell lysis using the lysis buffer provided in the Caspase 3 Assay Kit (REF.ab39383, Abcam). After centrifugation at 15,000*g*, the supernatant was collected, and reaction buffer along with caspase-3 substrate (DEVD-AFC) was added. Fluorescence intensity was measured using a fluorescence microplate reader with excitation at 400 nm and emission at 505 nm to assess caspase-3 activity (Varioskan LUX Multimode Microplate Reader, Thermo Fisher Scientific). Caspase-3 activity was evaluated by comparing the fluorescence intensity with that of the control group.

### Flow cytometry analysis.

After appropriate treatment, NSCLC cells were suspended in 500 μL of binding buffer, and then 5 μL of Annexin V-FITC and 10 μL of propidium iodide [Annexin V-FITC/PI Apoptosis Kit, REF.AP101-100, MultiSciences (Lianke) Biotech Co., Ltd] were added. The mixture was thoroughly mixed and incubated for 5 minutes at room temperature, protected from light. Finally, apoptotic cells were analyzed using flow cytometry. The antibody used in this experiment is part of the Annexin V-FITC/PI Apoptosis Kit.

### Immunoprecipitation.

NSCLC cells were treated and cell pellets were collected. Cells were then lysed in IP lysis buffer (REF.87788, Thermo Fisher Scientific) according to the manufacturer’s instructions. Protein concentration was determined using the BCA protein assay kit (REF.23225, Thermo Fisher Scientific), following the provided protocol. Then, 40 μL of A/G-agarose beads were prewashed and incubated with 2–4 mg of protein and 2 μg of the corresponding antibody overnight at 4°C. The next day, immunoprecipitated proteins were analyzed by WB to detect the target protein.

### IHC analysis.

The corresponding tissue sections were deparaffinized using xylene and ethanol and antigenically repaired using 10 mM sodium citrate powder solution. Subsequently, the sections were rinsed 3 times with distilled water and then incubated with 3% hydrogen peroxide (ready-to-use) for 10 minutes at room temperature to inactivate endogenous peroxidase and biotin. Next, the sections were blocked with goat serum for 10–30 minutes and then incubated with primary antibody at 4°C overnight. The next day, after washing the sections with PBS (3 times × 5 minutes), they were incubated with the secondary antibody for 45 minutes. Finally, DAB solution was added to develop the color, and after hematoxylin counterstaining, the positive staining was observed under a DM2000 microscope (Leica Microsystems).

### IF.

After the cells were treated according to the appropriate experimental protocol, the medium was aspirated and rinsed 3 times with PBS. Next, the cells were fixed using 4% paraformaldehyde for 10 minutes and then incubated with 0.3% Triton X-100 permeabilization solution for 20 minutes. Subsequently, the cells were washed with PBS (3 times × 5 minutes) and then sealed using a sealing solution, typically for 30 minutes. After sealing, the cells were incubated overnight at 4°C with the primary antibody and the corresponding fluorescence secondary antibody the next day. DAPI was used for nuclear staining. Finally, fluorescence microscopy (Eclipse Ti2 fluorescence microscope, Nikon Instruments Inc.) was used to observe the staining.

### Ubiquitination analysis.

NSCLC cell precipitates were collected by digestion and centrifugation at 500*g* after the cells were processed according to the appropriate experimental protocols. Cell lysates were lysed with RIPA lysate containing 1% SDS, sonicated, and heated in a metal bath at 95°C for 15 minutes. The cell lysate was centrifuged for 10–15 minutes (16,000*g*), the supernatant was aspirated, and the protein concentration was determined. The appropriate volume of supernatant protein was added to RIPA buffer containing 0.1% SDS (total volume 750 μL), followed by the appropriate antibodies and agarose beads, and coincubated overnight at 4°C. The supernatant was then incubated with the appropriate antibodies and agarose beads. The next day, the upsampling buffer was prepared according to the protocol.

### CHX assay.

NSCLC cells were pretreated with piperlongumine (4 μM) for 24 hours, and then NSCLC cells were treated with CHX (20 μg/mL) for 0, 1, 2, and 4 hours, respectively. Subsequently, WCE were collected to detect changes in Sp1 half-life using WB.

### Subcellular fraction isolation.

NSCLC cells were treated with piperlongumine for 24 hours, and the subcellular Protein Fractionation Kit (Thermo Fisher Scientific, REF.78840) and Mitochondria Isolation Kit (Thermo Fisher Scientific, REF.89874) were used to prepare proteins from different cellular compartments according to the standard instruction.

### qRT-PCR assay.

Cells were inoculated into 6-well plates at a density of 6 × 10^5^. When the cells reached 80%–90% confluence, the medium was aspirated and rinsed with PBS, TRIzol reagent (Invitrogen) was added to lyse the cells (500 μL/well), and the lysate was aspirated into 1.5 mL Eppendorf tubes. Subsequently, chloroform, isopropanol, and 75% ethanol were added sequentially to extract the RNA precipitate. After dissolving the RNA precipitate with 20–50 μL of enzyme-free water, the RNA concentration was determined, and 1,000 ng of RNA was taken for inversion (REF.K16215, Thermo Fisher Scientific). Finally, qRT-PCR experiments and data analysis were performed.

### In vivo tumor growth.

First, to demonstrate whether c-Met affects the in vivo tumorigenicity of osimertinib-resistant NSCLC cells, the in vivo model was prepared by injecting shCtrl and shc-Met HCC827OR cells into the right side of 6-week-old thymus-free nude mice (*n* = 5). Tumor growth volume was recorded (every 2 days) and calculated according to the formula (length × width^2^/2). When the tumor grew to 100 mm^3^, the mice were randomly divided into 4 groups: shCtrl+vehicle group, shCtrl+osimertinib group (5 mg/kg/d), shc-Met+vehicle group, and shc-Met+osimertinib group (5 mg/kg/d). Subsequently, when the tumor volume reached approximately 600 mm^3^, the mice were euthanized and the tumor weight was recorded. Tumor tissues were fixed in formaldehyde for IHC analysis. Next, it was investigated whether piperlongumine inhibited tumor growth of osimertinib-resistant NSCLC cells in vivo. HCC827OR and H1975OR cells were injected into nude mice (*n* = 6) to construct xenograft tumor models, respectively, and when the tumors grew up to 100 mm^3^, the mice were randomly divided into 3 groups: control group, low-piperlongumine group (5 mg/kg/2 days), and high-piperlongumine group (15 mg/kg/2 days). Subsequently, when the tumors grew to approximately 800 mm^3^, the mice were euthanized, tumor blocks were taken to record body weights, and the tumor tissues were fixed in formaldehyde for subsequent WB and IHC analysis. The blood of the mice was taken for the corresponding indexes. Finally, to further verify whether piperlongumine overcame the resistance of NSCLC cells to osimertinib, HCC827OR and H1975OR cells were injected into nude mice (*n* = 5) to construct a xenograft tumor model. When the tumors grew up to 100 mm^3^, the mice were randomly divided into 4 groups: control, osimertinib administration (2 mg/kg/d), piperlongumine administration (5 mg/kg/2 days), and coadministration groups (piperlongumine: 5 mg/kg/2 days; osimertinib: 2 mg/kg/d). Subsequently, when the tumors grew to approximately 800 mm^3^, the mice were euthanized, tumor blocks were taken to record body weights, and the tumor tissues were fixed in formaldehyde for subsequent WB and IHC analysis.

### Hematological analysis.

Blood samples of mice treated with different concentrations of piperlongumine were collected. The samples were further analyzed for changes in RBC, WBC, Hb, AST, ALT, and BUN indicators.

### Statistics.

The quantitative data from at least 3 independent experiments were statistically analyzed using GraphPad Prism software. Statistical significance was calculated using 2-tailed unpaired *t* test or 1-way ANOVA. Survival analysis was conducted using the log-rank test. *P* values of less than 0.05 were regarded as significant.

### Study approval.

All animal experiments were approved by the Institutional Animal Care and Use Committee, the Third Xiangya Hospital of Central South University (Changsha, China).

### Data availability.

Values for all data points in graphs are reported in the Supporting Data Values file.

## Author contributions

WL, QW, RW, and JL conceived and designed the study. RW, JL, and XY completed the experiments. WL and QW conducted data collation and statistical analysis. QW, RW, and JL completed the initial manuscript. WL and XY further checked and revised the manuscript. WL and QW provided funding support.

## Supplementary Material

Supplemental data

Unedited blot and gel images

Supplemental table 1

Supporting data values

## Version 1 03/24/2025

Electronic publication
