# Supplementary material for: Piperlongumine overcomes osimertinib resistance via governing ubiquitination-modulated Sp1 turnover
Source: JCI Insight. 2025 Mar 24;10(6):e186165. doi: 10.1172/jci.insight.186165 (PMC11949057; doi:10.1172/jci.insight.186165)
Supplement: Supplemental data [file jciinsight-10-186165-s033.pdf]

Supplementary figures

Figure S1

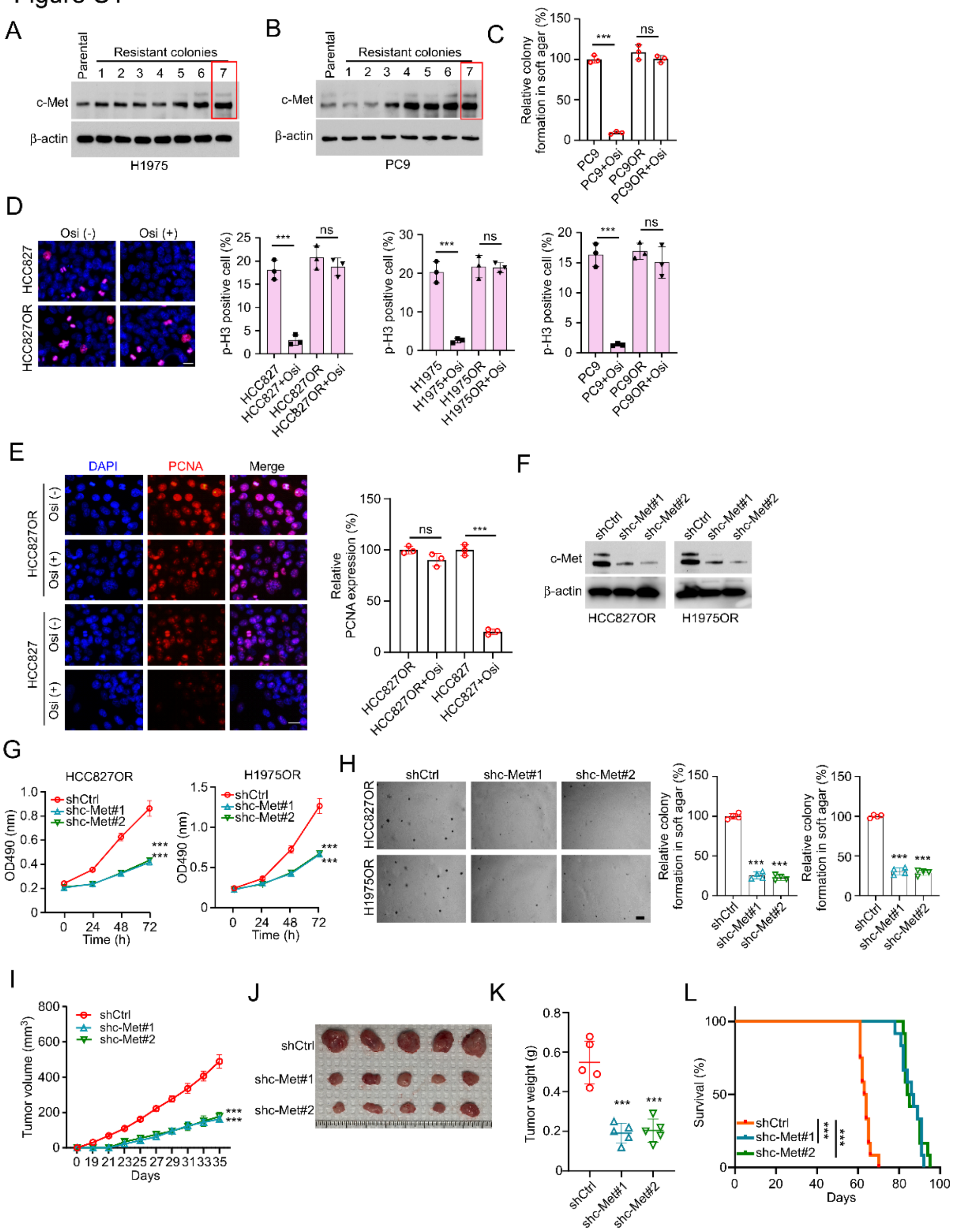

Figure S2

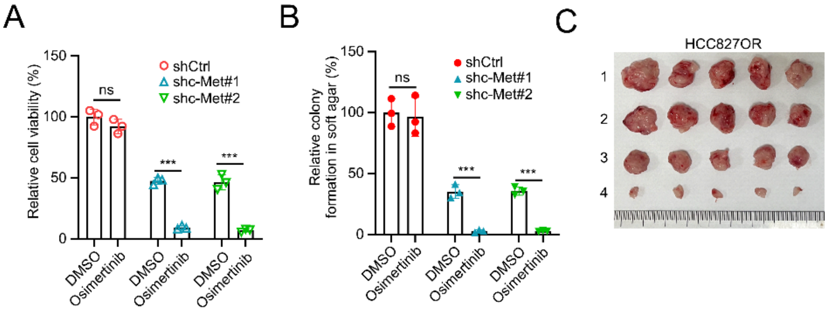

A
B
C
Relative cell viability (%)
150
100
50
0
ns
***
***
shCtrl
shc-Met#1
shc-Met#2
DMSO
Osimertinib
Relative colony formation in soft agar (%)
HCC827OR
1
2
3
4

Figure S3

A

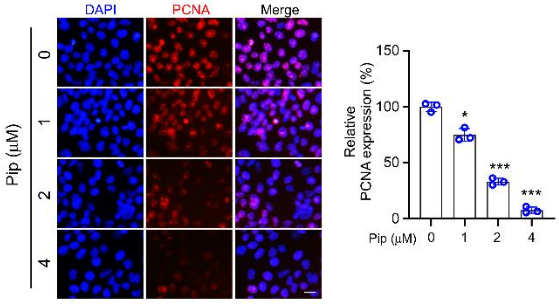
DAPI
PCNA
Merge
Pip (μM)
0
1
2
4
Relative PCNA expression (%)
150
100
50
0
*
***
***
Pip (μM)
0
1
2
4

Figure S4

A

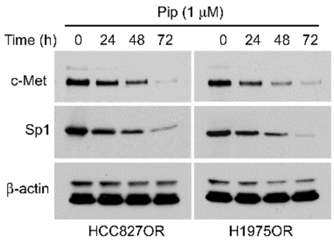

Pip (1 μM)
Time (h) 0 24 48 72 0 24 48 72
c-Met
Sp1
β-actin
HCC827OR
H1975OR

Figure S5

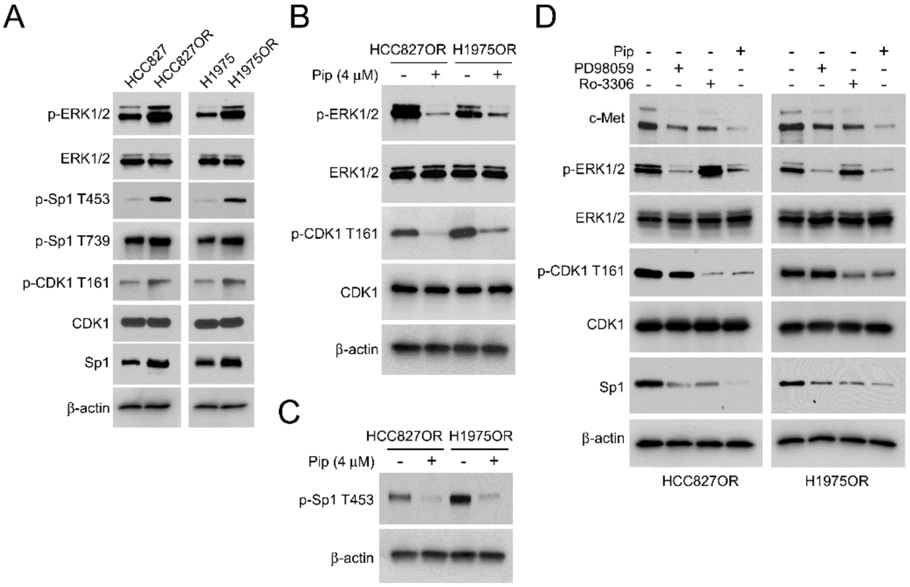
A
HCC827
HCC827OR
H1975
H1975OR
p-ERK1/2
ERK1/2
p-Sp1 T453
p-Sp1 T739
p-CDK1 T161
CDK1
Sp1
β-actin
B
HCC827OR
H1975OR
Pip (4 μM)
- + - +
p-ERK1/2
ERK1/2
p-CDK1 T161
CDK1
β-actin
C
HCC827OR
H1975OR
Pip (4 μM)
- + - +
p-Sp1 T453
β-actin
D
Pip
PD98059
Ro-3306
c-Met
p-ERK1/2
ERK1/2
p-CDK1 T161
CDK1
Sp1
β-actin
HCC827OR
H1975OR

Figure S6

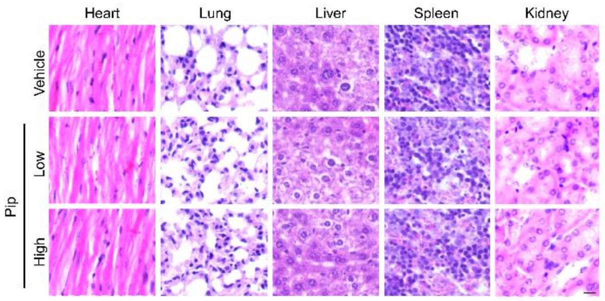

Supplementary figure legends

Figure S1. The critical role of c-Met in maintaining tumorigenicity of osimertinib-resistant cells.

A-B. Immunoblotting was used to analyze the expression of c-Met in 7 clones of H1975 (A) and PC9 (B) cells and their parental cells. C. Colony formation ability of PC9OR

cells was detected after treatment with osimertinib using soft agar assay. ns, not statistically significant. n=3, ***p<0.001. D. Immunofluorescence (IF) analysis of p-H3 Ser10 positive cells in osimertinib-treated PC9/PC9OR, HCC827/HCC827OR, and H1975/H1975OR cells. Scale bar, 10 μm. ns, not statistically significant. n=3, ***p<0.001. E. IF analysis of PCNA expression in osimertinib-treated HCC827/HCC827OR cells. Scale bar, 10 μm. ns, not statistically significant. n=3, ***p<0.001. F-H. The protein levels of c-Met were detected by WB analysis (F), cell viability level was tested by MTS assay (G, n=3, ***p<0.001.), and colony-forming ability was measured by soft agar assay (H, n=3, Scale bar 200 μm. ***p<0.001.). I-L. Xenograft tumor models were constructed using shc-Met HCC827OR cells, and tumor volume (I, ***p<0.001.), tumor mass (J), and tumor weight (K, ***p<0.001.) were detected. The survival analysis of tumor-bearing mice with shCtrl or shc-Met xenograft tumors by Kaplan-Meier method (L, n=12 mice per group. ***p<0.001.) . Comparisons were performed by using by 1-way ANOVA test (C-E, G-I and K) and log-rank (Mantel-Cox) test (L). Data are presented as the mean ± SD (C-E, G-I and K).

Figure S2. c-Met knockdown restored the sensitivity of PC9OR to osimertinib.
A-B. The shCtrl and shc-Met PC9OR cells were treated with osimertinib or DMSO. MTS assay (A) and soft agar assay (B) were used to analyze the cell viability and colony formation ability of PC9OR cells, respectively. n=3, ***p<0.001. C. shc-Met HCC827OR cells were utilized to construct a xenograft transplantation tumor model and analyzed the effects of c-Met deletion and administration of osimertinib treatment on tumor mass. n=5. Comparisons were performed by using 1-way ANOVA test (A and B). Data are presented as the mean ± SD (A and B).

Figure S3. Piperlongumine inhibited proliferation in HCC827OR Cells.

A. IF analysis of PCNA expression in Piperlongumine-treated HCC827OR cells. Scale bar, 10 μm. n=3. *p<0.05. ***p<0.001. Comparisons were performed by using 1-way ANOVA test. Data are presented as the mean ± SD.

Figure S4. Extended Piperlongumine treatment significantly inhibits Sp1 and c-Met.

A. After treating HCC827OR and H1975OR cells with Piperlongumine for different time periods, the protein expression levels of c-Met and Sp1 were detected by IB analysis.

Figure S5. Role of ERK1/2 and CDK1 in the regulation of Sp1 in osimertinib-resistant cells.

A. WCE from HCC827/HCC827OR and H1975/H1975OR cells were collected and subjected to IB analysis. B-C. After treating HCC827OR and H1975OR cells with Piperlongumine for 24 h, cells were collected and subjected to IB analysis. D. After treating HCC827OR and H1975OR cells with Piperlongumine, PD98059 (a specific inhibitor of ERK1/2), or Ro-3306 (a specific inhibitor of CDK1) individually for 24 h, WCE was collected and subjected to IB analysis.

Figure S6. Piperlongumine treatment did not damage the vital organs of mice.

A. HE staining of heart, lung, liver, spleen and kidney sections of HCC827OR-derived tumor-bearing mice treated with vehicle, osimertinib, Piperlongumine, or combination. Scale bar, 10 μm.
