# Supplementary material for: Piperlongumine overcomes osimertinib resistance via governing ubiquitination-modulated Sp1 turnover
Source: JCI Insight. 2025 Mar 24;10(6):e186165. doi: 10.1172/jci.insight.186165 (PMC11949057; doi:10.1172/jci.insight.186165)

Full gel for Figure 1

E

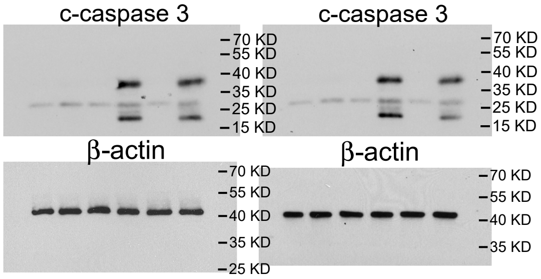

Full gel for Figure 3

C

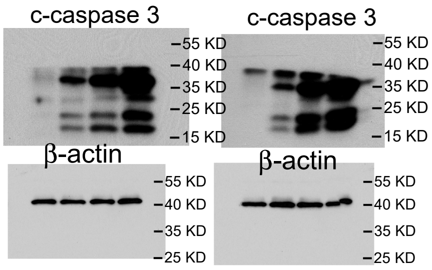

G

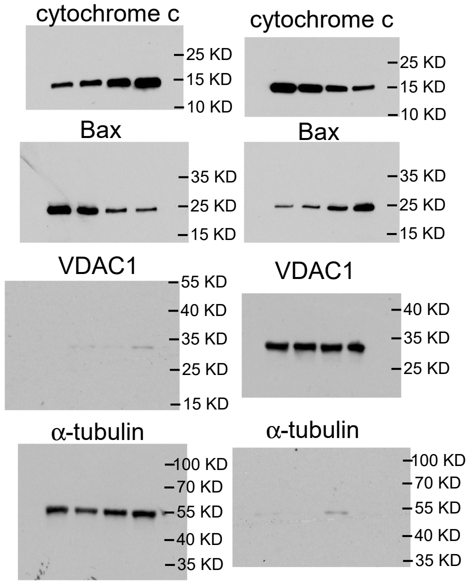

Full gel for Figure 4

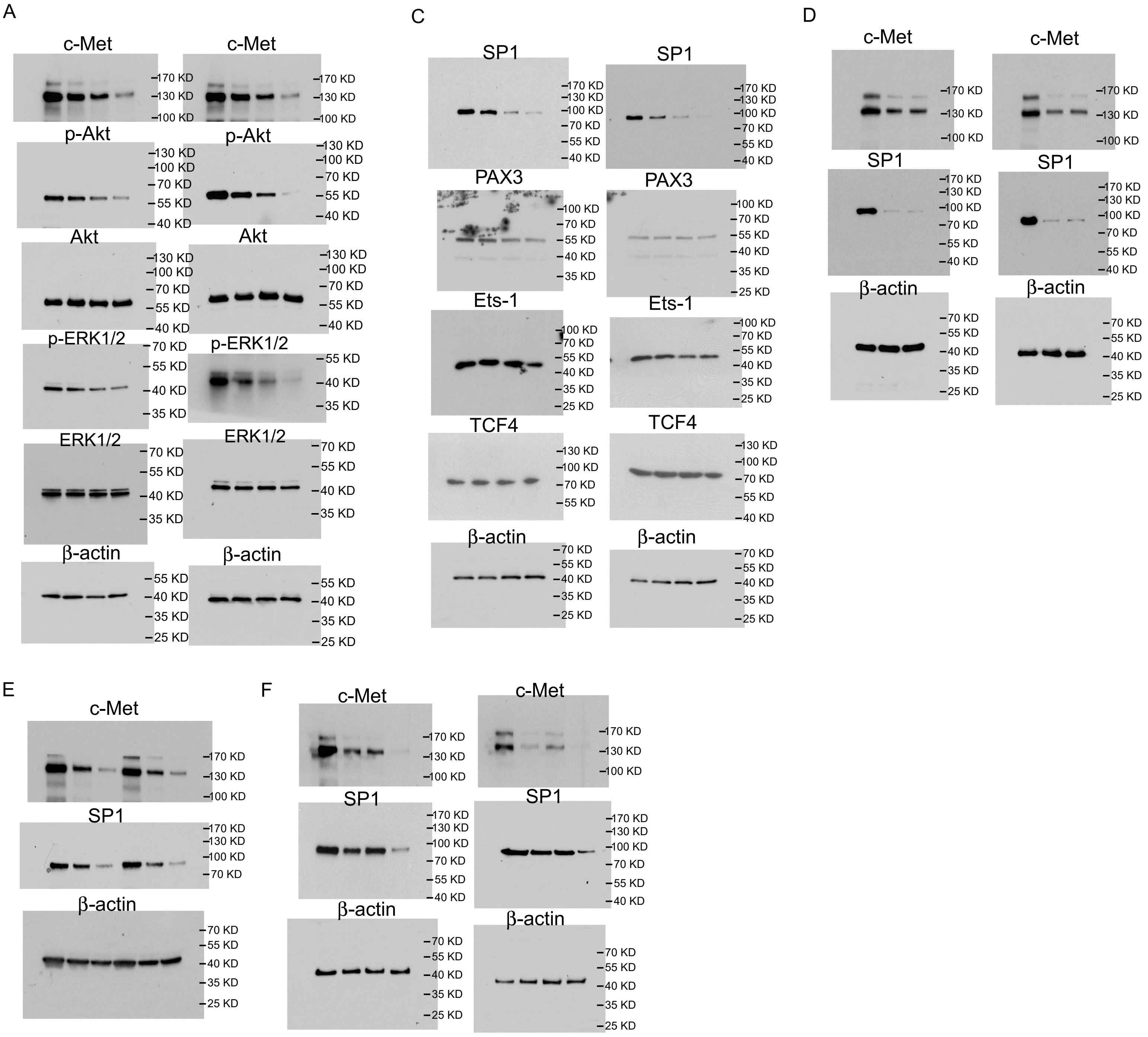

Full gel for Figure 5

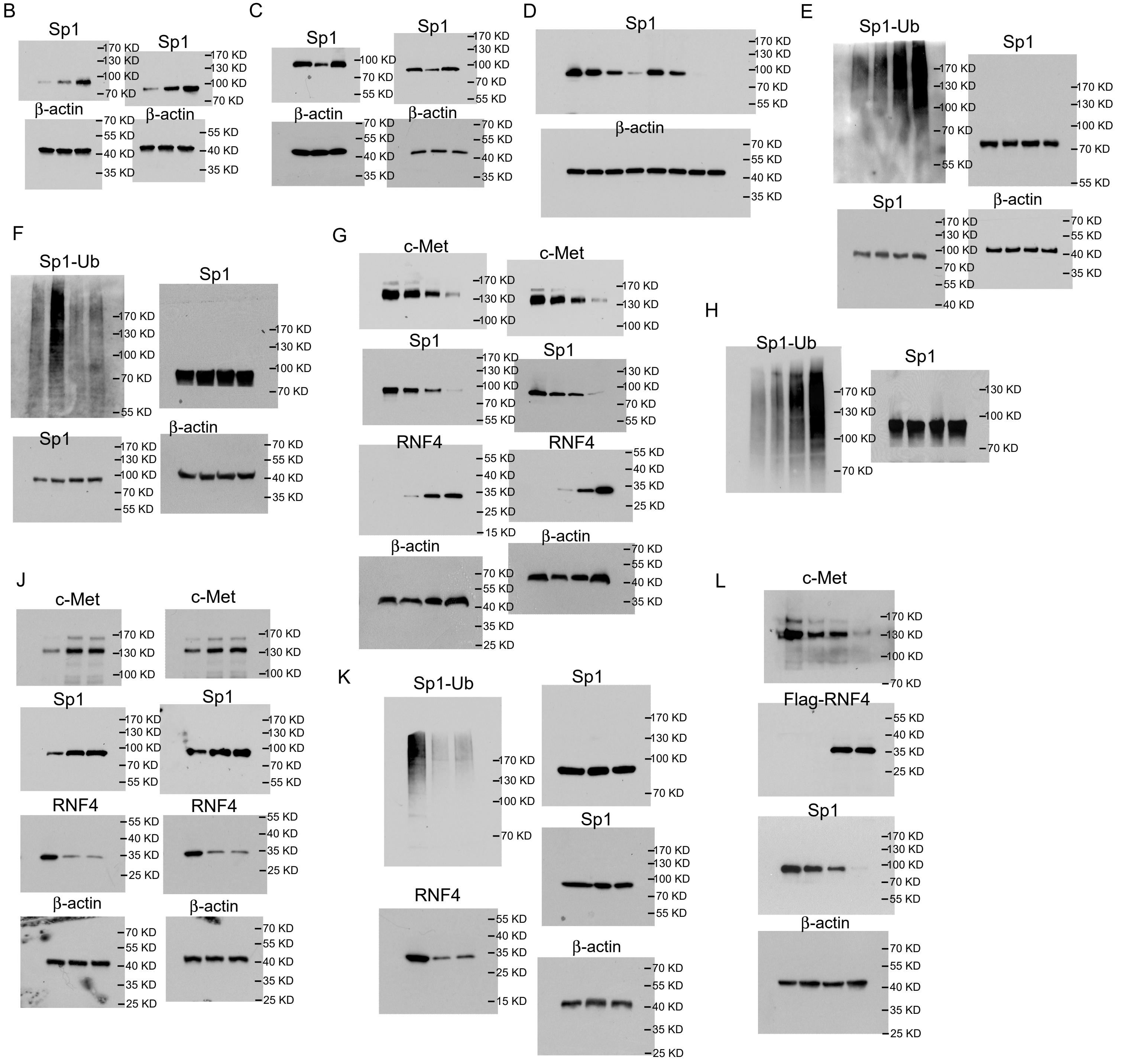

Full gel for Figure 6

A

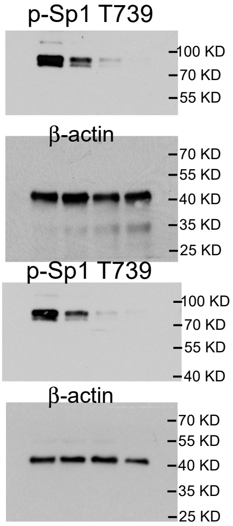

B

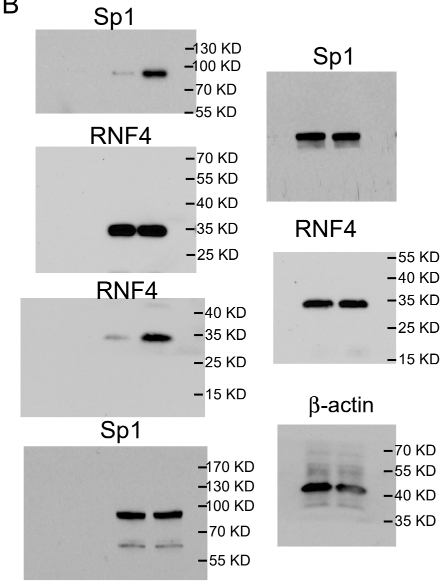

C

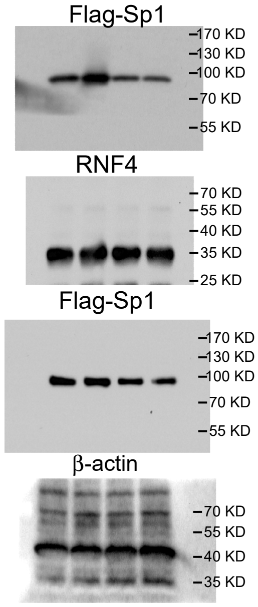

D

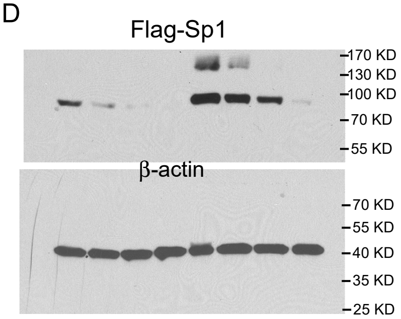

E

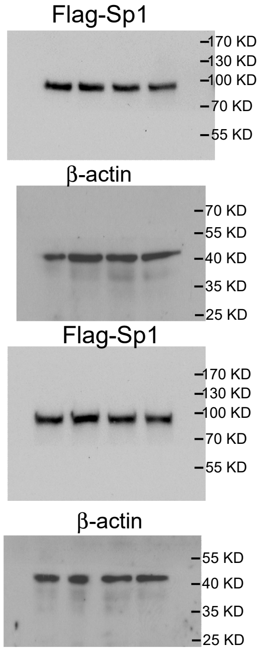

F

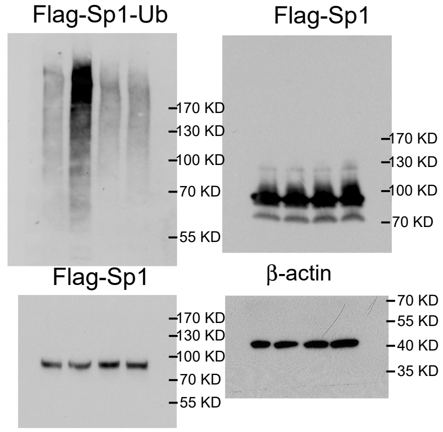

G

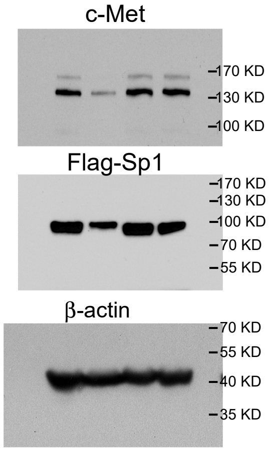

Full gel for Figure 8

D

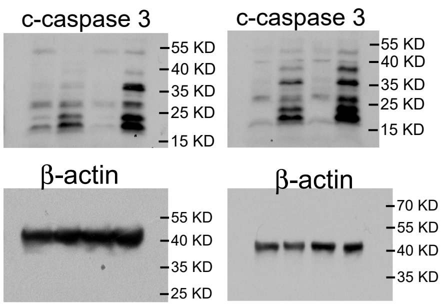

Full gel for Supplementary Figure 1

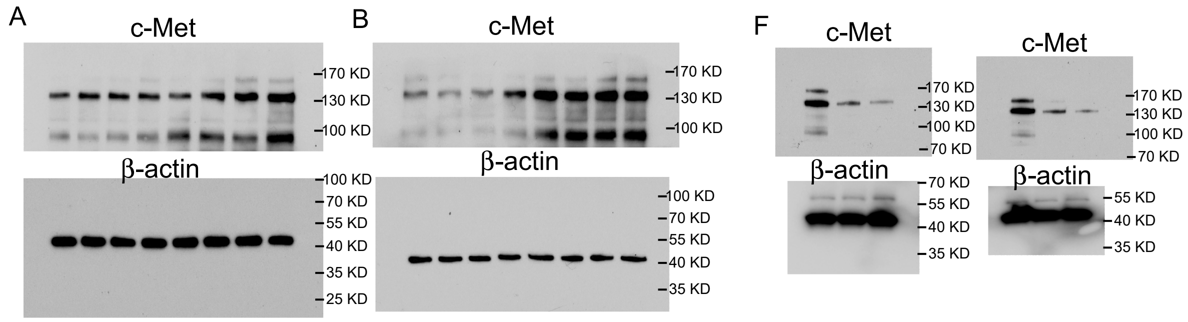

Full gel for Supplementary Figure 4

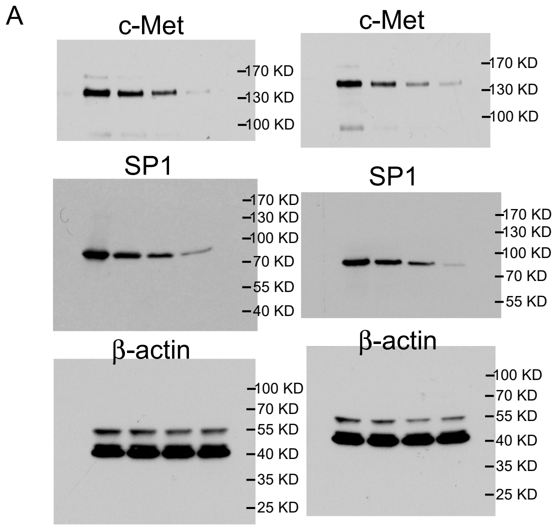

Full gel for Supplementary Figure 5

A

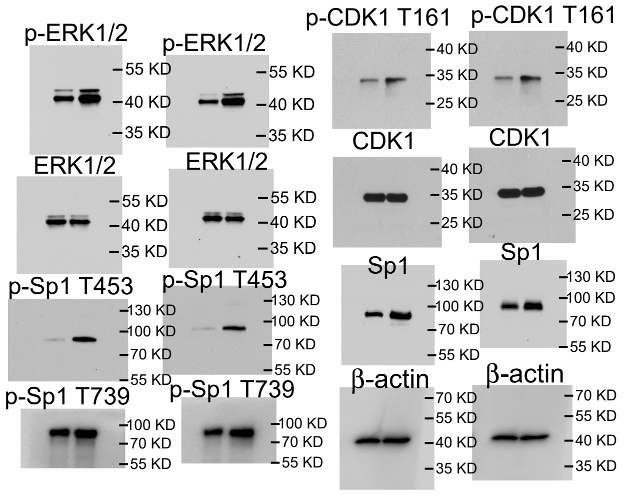

B

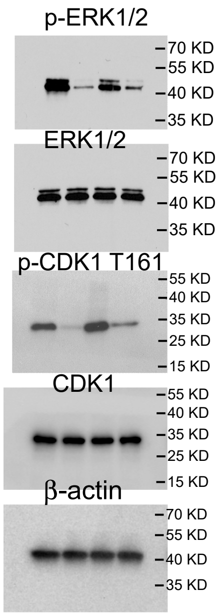

C

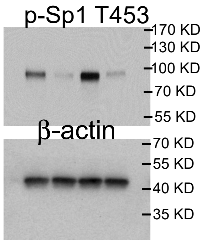

D

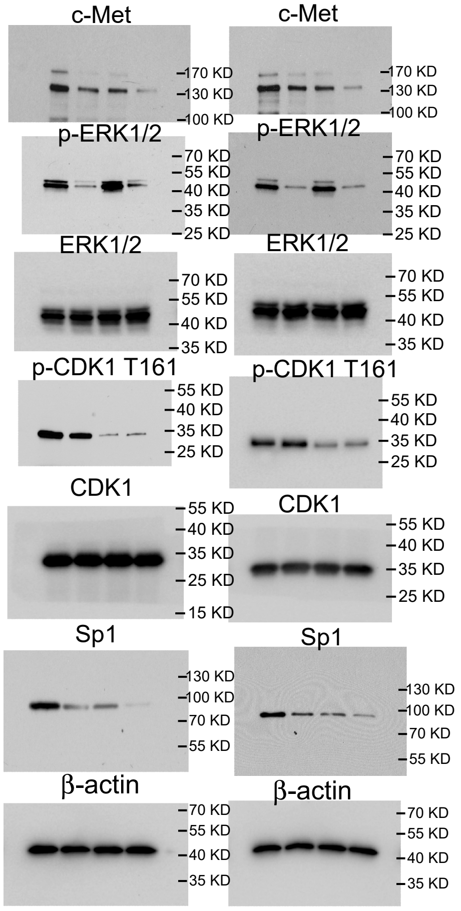

Supplement: Unedited blot and gel images [file jciinsight-10-186165-s034.pdf]
